# The influence of receptor expression and clinical subtypes on baseline [18F]FDG uptake in breast cancer: systematic review and meta-analysis

**DOI:** 10.1186/s13550-023-00953-y

**Published:** 2023-01-23

**Authors:** Cornelis M. de Mooij, Roxanne A. W. Ploumen, Patty J. Nelemans, Felix M. Mottaghy, Marjolein L. Smidt, Thiemo J. A. van Nijnatten

**Affiliations:** 1 Department of Radiology and Nuclear Medicine, Maastricht University Medical Centre+, P.O. Box 5800, 6202 AZ Maastricht, The Netherlands; 2 Department of Surgery, Maastricht University Medical Centre+, Maastricht, The Netherlands; 3 GROW – School for Oncology and Reproduction, Maastricht University Medical Centre+, Maastricht, The Netherlands; 4 Department of Epidemiology, Maastricht University Medical Centre+, Maastricht, The Netherlands; 5 Department of Nuclear Medicine, University Hospital RWTH Aachen University, Aachen, Germany

**Keywords:** Breast cancer, Immunohistochemistry, Clinical subtypes, [18F]FDG PET, Systematic review, Meta-analyses

## Abstract

**Background:**

To quantify the relationship between [18F]FDG uptake of the primary tumour measured by PET-imaging with immunohistochemical (IHC) expression of ER, PR, HER2, Ki-67, and clinical subtypes based on these markers in breast cancer patients.

**Methods:**

PubMed and Embase were searched for studies that compared SUV_max_ between breast cancer patients negative and positive for IHC expression of ER, PR, HER2, Ki-67, and clinical subtypes based on these markers. Two reviewers independently screened the studies and extracted the data. Standardized mean differences (SMD) and 95% confidence intervals (CIs) were estimated by using DerSimonian-Laird random-effects models. *P* values less than or equal to 5% indicated statistically significant results.

**Results:**

Fifty studies were included in the final analysis. SUV_max_ is significantly higher in ER-negative (31 studies, SMD 0.66, 0.56–0.77, *P* < 0.0001), PR-negative (30 studies, SMD 0.56; 0.40–0.71, *P* < 0.0001), HER2-positive (32 studies, SMD − 0.29, − 0.49 to − 0.10, *P* = 0.0043) or Ki-67-positive (19 studies, SMD − 0.77; − 0.93 to − 0.61, *P* < 0.0001) primary tumours compared to their counterparts. The majority of clinical subtypes were either luminal A (LA), luminal B (LB), HER2-positive or triple negative breast cancer (TNBC). LA is associated with significantly lower SUV_max_ compared to LB (11 studies, SMD − 0.49, − 0.68 to − 0.31, *P* = 0.0001), HER2-positive (15 studies, SMD − 0.91, − 1.21 to − 0.61, *P* < 0.0001) and TNBC (17 studies, SMD − 1.21, − 1.57 to − 0.85, *P* < 0.0001); and LB showed significantly lower uptake compared to TNBC (10 studies, SMD − 0.77, − 1.05 to − 0.49, *P* = 0.0002). Differences in SUV_max_ between LB and HER2-positive (9 studies, SMD − 0.32, − 0.88 to 0.24, *P* = 0.2244), and HER2-positive and TNBC (17 studies, SMD − 0.29, − 0.61 to 0.02, *P* = 0.0667) are not significant.

**Conclusion:**

Primary tumour SUV_max_ is significantly higher in ER-negative, PR-negative, HER2-positive and Ki-67-positive breast cancer patients. Luminal tumours have the lowest and TNBC tumours the highest SUV_max_. HER2 overexpression has an intermediate effect.

**Supplementary Information:**

The online version contains supplementary material available at 10.1186/s13550-023-00953-y.

## Background

Immunohistochemical (IHC) detection of estrogen receptor (ER), progesterone receptor (PR) and human epidermal growth factor receptor 2 (HER2) is the foundation of clinical subtyping of breast cancer since it selects targets for endocrine or HER2-targeted therapy [1–3]. In addition, gene expression profiling (GEP) studies have identified at least four intrinsic breast cancer subtypes that more accurately capture the diversity of breast cancer [4, 5]. Surrogate intrinsic subtypes have been defined which can be approximated using IHC determination of ER, PR, HER2 and Ki-67 [6–8]. To date, clinical subtyping using IHC has near exclusive use in contemporary practice.

Positron emission-tomography (PET) using [18F]-fluorodeoxyglucose ([18F]FDG) is a widely accepted imaging modality in breast cancer that is nowadays mostly used in combination with computed tomography (PET/CT) or magnetic resonance imaging (PET/MRI) for anatomic correlation. While mainly used for initial staging in patients with locally advanced or suspected recurrent breast cancer, it has also been thoroughly investigated for its ability to predict and detect response to neoadjuvant systemic therapy (NST) and to predict prognosis [9–11]. In practice, [18F]FDG uptake is predominantly expressed using maximum standardized uptake values (SUV_max_).

Previous studies report a correlation of [18F]FDG uptake with tumour aggressiveness, with increased SUV_max_ in primary breast tumours that are ER-negative, PR-negative, HER2-positive or Ki-67-positive [12–14]. Studies investigating the difference in [18F]FDG uptake between clinical subtypes have found a similar pattern with relatively low SUV_max_ in subtypes including ER and PR, and high SUV_max_ for subtypes including HER2 or that are triple negative [15, 16]. To date, no meta-analysis has investigated or quantified the relative difference in SUV_max_ between IHC expression of ER, PR, HER2, Ki-67, and clinical subtypes based on these markers.

Therefore, the aim of the present study is to perform a systematic review and meta-analysis to investigate and quantify the association between [18F]FDG uptake expressed as SUV_max_ and IHC expression of ER, PR, HER2, Ki-67, and clinical subtypes based on these markers.

## Methods

The full description of the methods can be obtained in Additional file 1 (Tables S1–S2). To be eligible for the meta-analysis, a study had to fulfill the following inclusion criteria: patients with invasive breast cancer, [18F]FDG uptake expressed as SUV_max_ and measured on the primary tumour before any therapy, comparison of [18F]FDG uptake between patients negative and positive for IHC expression of ER, PR, HER2, or Ki-67, and between clinical subtypes based on the IHC expression of these markers. Data on the number of patients, mean and standard deviation (SD) of SUV_max_ of patients negative and positive for IHC expression of ER, PR, HER2, Ki-67, and clinical subtypes based on these markers, was extracted. Study quality was assessed by using the Quality Assessment of Diagnostic Accuracy Studies (QUADAS)-2 tool. For the meta-analysis, the primary summary statistic was the standardized mean difference (SMD) with 95% confidence intervals (CIs) using Hedges’ g correction for small study samples. The primary analyses were based on studies which presented mean [18F]FDG uptake with SD. Sensitivity analyses also included studies which presented median [18F]FDG uptake with (interquartile) range which were transformed to mean and SD. Lastly, Egger’s regression test was used to identify small-study effects.

## Results

### Study characteristics and QUADAS-2

**Fig. 1:**
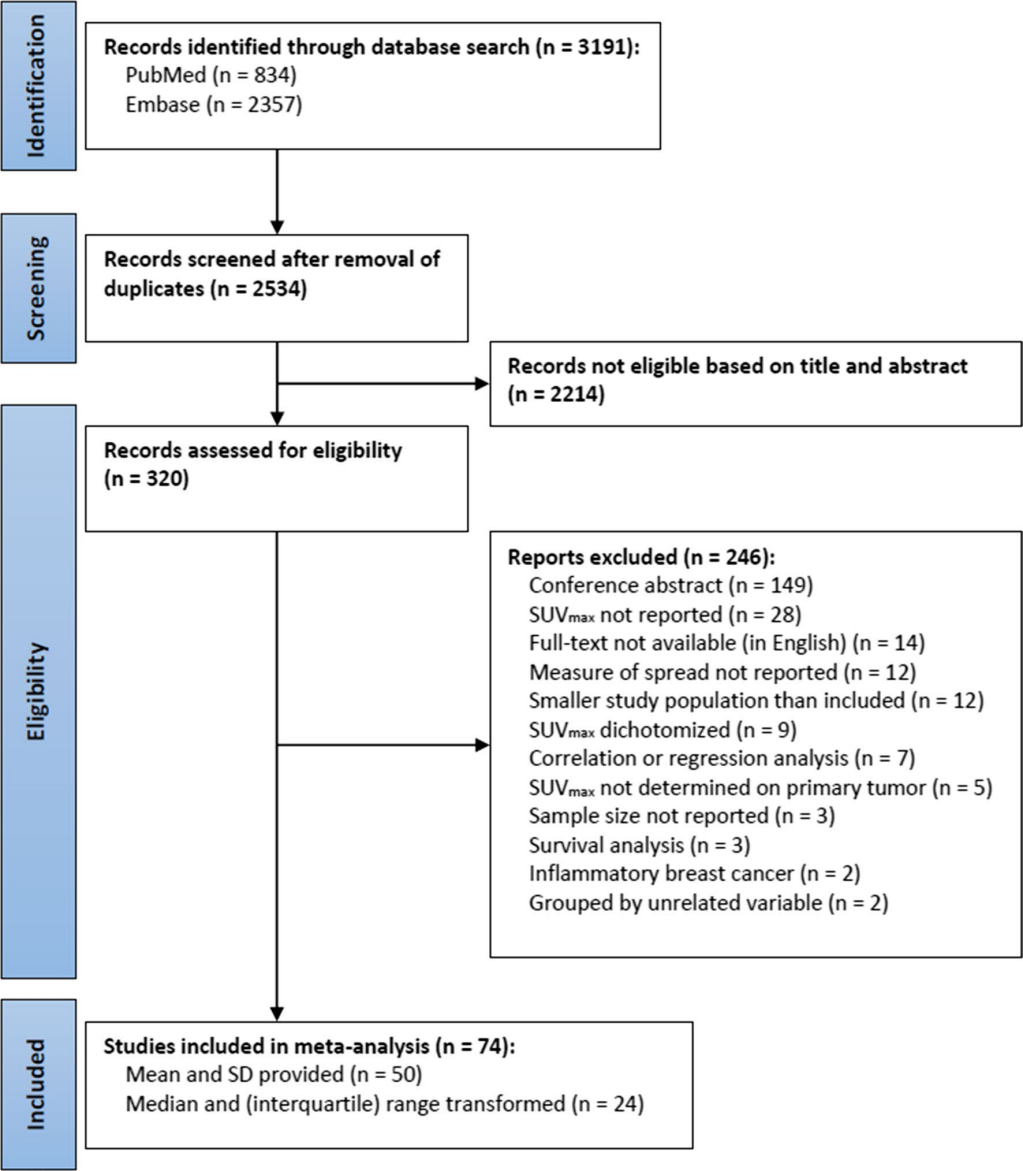
PRISMA flow diagram of the study selection

Figure 1 shows the search pattern and selection of articles at each step. Of the 74 included studies the means and SDs were provided in 50 [12, 14, 16–63]. In the remaining 24 studies the means and SDs were transformed from the provided medians and (interquartile) ranges [13, 64–87]. An overview of the characteristics of included studies as well as the [18F]FDG PET characteristics is provided in Additional file 2 (Tables S3–S4). The number of patients, mean and SD of each individual study for negative and positive IHC expression of ER, PR, HER2, Ki-67, and of clinical subtypes based on these markers, is provided in Additional file 2 (Tables S5–S11).

### Quality of included studies

**Fig. 2:**
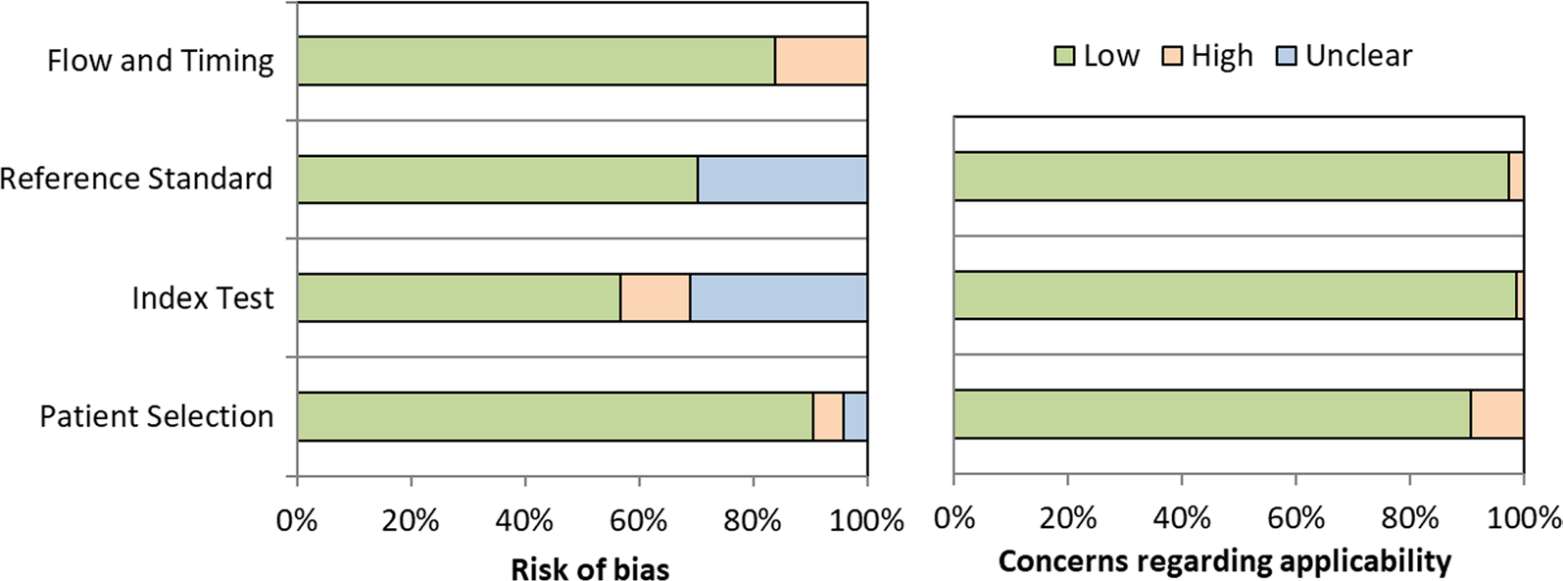
Methodological quality of included studies

Risk of bias for patient selection originated from poor reporting of in- and exclusion criteria in three studies and the use of case–control designs in another three studies. For the index test, there was an unclear risk of bias in 26 studies since it was not reported who reviewed the PET images or performed SUV_max_ measurements, and a high risk of bias in 8 studies since no harmonization of PET-data was performed while using multiple PET-devices. With regard to the reference standard, 22 studies did not provide criteria for receptor positivity or subtypes. Lastly, high risk of bias in flow and timing existed in 8 studies since not all patients were included in the final analysis without providing valid reasons. In general, applicability concerns are low, meaning that the patient selection, index test and reference standard of the included studies match the review question. Figure 2 visualizes the risk of bias and applicability concerns and additional information on methodologic quality of individual studies is provided in Additional file 2 (Table S12).

### Association between [18F]FDG uptake and receptor status

Table 1 displays the estimates of the SMD with 95% CIs as measure for the difference in [18F]FDG uptake between negative versus positive IHC expression of ER, PR, HER2 and Ki-67. The primary analyses show that the SUV_max_ is significantly higher in ER-negative (SMD 0.66, *P* < 0.0001), PR-negative (SMD 0.56, *P* < 0.0001), HER2-positive (SMD − 0.29, *P* = 0.0043) or Ki-67-positive (SMD − 0.77, *P* < 0.0001) primary tumours compared to their counterparts.

**Table 1:** Estimates of the SMD as summary measure for the difference in [18F]FDG (SUV_max_) uptake between negative versus positive IHC expression of ER, PR, HER2, and Ki-67

Receptor	Studies	Patients		Meta-analysis				Subgroup	Egger
	No	Negative	Positive	*I*^2^ (%)	SMD	95% CI	*P*	*P*	*P*
Primary analyses									
ER	31	1659	3777	48.0	0.66	0.56, 0.77	< 0.0001	–	0.6530
PR	30	2043	2788	71.6	0.56	0.40, 0.71	< 0.0001	–	0.7426
HER2	32	4035	1664	80.0	− 0.29	− 0.49, − 0.10	0.0043	–	0.4726
Ki-67	19	1720	2186	57.8	− 0.77	− 0.93, − 0.61	< 0.0001	–	0.8838
Sensitivity analyses									
ER	47	2181	5256	43.1	0.67	0.59, 0.75	< 0.0001	0.7980	0.7934
PR	46	2764	4171	66.4	0.54	0.42, 0.65	< 0.0001	0.6328	0.8925
HER2	49	5602	2221	74.5	− 0.30	− 0.43, − 0.16	< 0.0001	0.9322	0.6184
Ki-67	28	2187	3028	51.0	− 0.75	− 0.87, − 0.64	< 0.0001	0.5364	0.7299

Data derived from the primary and sensitivity analyses are presented

CI, confidence interval; ER, estrogen receptor; HER2, human epidermal growth factor 2 receptor; PR, progesterone receptor; SMD, standardized mean difference

### Association between [18F]FDG uptake and surrogate intrinsic subtypes

The estimates of the SMD with 95% CIs as measure for the difference in [18F]FDG uptake between surrogate intrinsic subtypes based on recommendations from the St. Gallen conferences is displayed in Table 2. The primary analyses reveal that LA was associated with significantly lower SUV_max_ than LB (SMD − 0.49, *P* = 0.0001), LB HER2-negative (SMD − 0.68, *P* = 0.0021), LB HER2-positive (SMD − 0.72, *P* = 0.0089), HER2-positive (SMD − 0.91, *P* < 0.0001) and TNBC (SMD − 1.21, *P* < 0.0001); LB significantly lower than TNBC (SMD − 0.77, *P* = 0.0002); LB HER2-negative significantly lower than TNBC (SMD − 0.58, *P* = 0.0177); LB HER2-positive significantly lower than HER2-positive (SMD − 0.22, *P* = 0.0457); and TNBC significantly higher than non-TNBC (SMD 0.56, *P* < 0.0001). While the sensitivity analyses did not reveal a difference in the direction of the meta-analyses, the size and 95% CIs of the SMDs did differ significantly for the comparison of LA with LB HER2-negative (*P* = 0.0213) and of TNBC versus non-TNBC (*P* = 0.0015) when including transformed medians and (interquartile) ranges.

**Table 2:** Estimates of the SMD as summary measure for the difference in [18F]FDG (SUV_max_) uptake between St. Gallen surrogate intrisic subtypes

Comparisons	Studies	Patients		Meta-analysis				Subgroup	Egger
	No			*I*^2^ (%)	SMD	95% CI	*P*	*P*	*P*
Primary analyses									
LB versus									
LB	11	1022	487	29.8	− 0.49	− 0.68, − 0.31	0.0001	–	0.8191
LBHER2−	6	234	373	32.5	− 0.68	− 0.97, − 0.38	0.0021	–	0.4378
LBHER2+	6	234	142	54.2	− 0.72	− 1.17, − 0.28	0.0089	–	0.2371
HER2+	15	1024	290	56.4	− 0.91	− 1.21, − 0.61	< 0.0001	–	0.6148
TNBC	17	1054	440	63.8	− 1.21	− 1.57, − 0.85	< 0.0001	–	0.7310
LB versus									
HER2+	9	369	185	61.2	− 0.32	− 0.88, 0.24	0.2244	–	0.8729
TNBC	10	405	279	36.0	− 0.77	− 1.05, − 0.49	0.0002	–	0.5091
LBHER2− versus									
LBHER2+	6	373	142	66.0	− 0.02	− 0.52, 0.48	0.9316	–	0.1000
HER2+	6	373	105	51.7	− 0.33	− 0.81, 0.14	0.1305	–	0.4260
TNBC	6	373	157	49.5	− 0.58	− 1.02, − 0.15	0.0177	–	0.7121
LBHER2+ versus									
HER2+	7	189	129	0.0	− 0.22	− 0.43, − 0.01	0.0457	–	0.3950
TNBC	8	220	198	60.7	− 0.45	− 0.98, 0.08	0.0864	–	0.2661
HER2+ versus									
TNBC	17	326	492	58.1	− 0.29	− 0.61, 0.02	0.0667	–	0.6702
TNBC versus									
Non-TNBC	13	283	1157	0.0	0.56	0.41, 0.70	< 0.0001	–	0.1236
Sensitivity analyses									
LA versus									
LB	16	1361	1103	58.1	− 0.46	− 0.64, − 0.28	< 0.0001	0.6555	0.4305
LBHER2−	7	309	522	54.9	− 0.60	− 0.90, − 0.31	0.0025	0.0213	0.1428
LBHER2+	7	309	176	46.4	− 0.71	− 1.07, − 0.36	0.0026	0.7466	0.3720
HER2+	21	1438	706	59.0	− 0.85	− 1.08, − 0.62	< 00,001	0.4906	0.6625
TNBC	24	1499	865	76.5	− 1.18	− 1.48, − 0.88	< 0.0001	0.7134	0.6259
LB versus									
HER2-pure	14	985	579	58.1	− 0.37	− 0.67, − 0.08	0.0170	0.5380	0.3568
TNBC	15	1021	614	49.4	− 0.75	− 0.95, − 0.55	< 0.0001	0.7621	0.2725
LBHER2− versus									
LBHER2+	7	522	176	65.3	− 0.09	− 0.52, 0.33	0.6078	0.1151	0.0428
HER2+	7	522	139	42.4	− 0.37	− 0.73, − 0.01	0.0454	0.6619	0.3687
TNBC	7	522	233	45.1	− 0.53	− 0.86, − 0.21	0.0073	0.3633	0.4680
LBHER2+ versus									
HER2+	8	223	151	0.0	− 0.17	− 0.37, 0.02	0.0745	0.3306	0.3426
TNBC	9	254	274	62.3	− 0.37	− 0.84, 0.10	0.1103	0.0792	0.1436
HER2+ versus									
TNBC	24	754	916	45.2	− 0.25	− 0.45, − 0.06	0.0130	0.9067	0.3980
TNBC versus									
Non-TNBC	19	379	1516	40.6	0.73	0.54, 0.90	< 0.0001	0.0015	0.0526

Data derived from the primary and sensitivity analyses are presented

CI, confidence interval; HER2, human epidermal growth factor 2 receptor; LA, luminal A; LB, luminal B; SMD, standardized mean difference; TNBC, triple negative breast cancer

### Association between [18F]FDG uptake and clinical subtypes according to a simplified classification

Table 3 displays the estimates of the SMD with 95% CIs as measure for the difference in [18F]FDG uptake between clinical subtypes according to a simplified classification which classified patients into three groups (i.e. ER-positive/HER2-negative, HER2-positive, and TNBC). The primary analyses reveal that SUV_max_ was significantly lower in ER-positive/HER2-negative than in HER2-positive (SMD − 0.34, *P* = 0.0070) or in TNBC (SMD − 0.89, *P* = 0.0008) and significantly lower in HER2-positive than in TNBC (SMD − 0.54, *P* = 0.0193).

**Table 3:** Estimates of the SMD as summary measure for the difference in [18F]FDG (SUV_max_) uptake between clinical subtypes according to a simplified classification

Comparisons	Studies	Patients		Meta-analysis				Subgroup	Egger
	No			*I*^2^ (%)	SMD	95% CI	*P*	*P*	
Primary analyses									
ER+/HER2− versus HER2+	5	755	302	0.0	− 0.34	− 0.53, − 0.16	0.0070	–	0.2633
ER+/HER2− versus TNBC	6	814	309	56.1	− 0.89	− 1.20, − 0.58	0.0008	–	0.0247
HER2+ versus TNBC	5	302	291	64.7	− 0.54	− 0.93, − 0.14	0.0193	–	0.3140
Sensitivity analyses									
ER+/HER2− versus HER2+	8	1153	416	30.9	− 0.38	− 0.56, − 0.20	0.0016	0.3985	0.7816
ER+/HER2− versus TNBC	9	1212	424	43.0	− 0.91	− 1.10, − 0.73	< 0.0001	0.3252	0.0246
HER2+ versus TNBC	8	416	406	22.9	− 0.50	− 0.76, − 0.24	0.0025	0.6884	0.5186

Data derived from the primary and sensitivity analyses are presented

CI, confidence interval; ER, estrogen receptor; HER2, human epidermal growth factor receptor 2; SMD, standardized mean difference; TNBC, triple negative breast cancer

## Discussion

The results of this systematic review and meta-analysis indicate that there are substantial differences in [18F]FDG uptake expressed as SUV_max_ of the primary tumour between negative and positive IHC expression of ER, PR, HER2, Ki-67, and between clinical subtypes based on these markers. The pooled SMD estimated significantly increased SUV_max_ in tumours that are ER-negative, PR-negative, HER2-positive and Ki-67-positive. Clinical subtypes based on these markers follow the same pattern with lower SUV_max_ in luminal subtypes including ER and PR, and higher uptake in TNBC. HER2 overexpression and associated subtypes have an intermediate effect, with significantly higher uptake compared to LA and LB HER2-positive, similar uptake compared to LB and LB HER2-negative, and insignificantly lower uptake compared to TNBC.

The effect of IHC expression of each separate marker (i.e. ER, PR, HER2 and Ki-67) on [18F]FDG uptake can partially be explained by both the interrelations as well as the underlying differences in confounding clinicopathologic factors. Proliferation marker Ki-67, having the single largest effect on [18F]FDG uptake in our meta-analysis, is closely related to histological or nuclear grading and proliferative, poorly differentiated tumours are more common in ER-negative, PR-negative and HER2-positive tumours [88, 89]. In addition, tumour size has an independent effect on [18F]FDG uptake and ER-negative, PR-negative, HER2-positive, and Ki-67-positive tumours are associated with larger sizes [14, 88, 90]. This difference is further increased by an underestimation of [18F]FDG uptake in smaller tumours due to partial volume effects [91]. Lastly, invasive lobular carcinoma is associated with lower [18F]FDG uptake and is especially common in ER-positive, PR-positive and Ki-67-negative tumours [14, 92].

Clinical subtyping provides a more sophisticated classification of breast cancer compared to the separate evaluation of IHC markers. Decreased [18F]FDG uptake in luminal tumours can be attributed to ER and PR expression, with an increase in avidity in case of HER2-positivity as displayed by the increase in [18F]FDG uptake in LB and HER2-positive subtypes. Analogous to separate markers, [18F]FDG uptake closely mimicks the degree of proliferation and differentiation with a gradual increase in both [18F]FDG uptake as well as Ki-67 labeling index and poorly differentiated tumours from LA, LB, HER2-positive to TNBC [93, 94]. Paradoxically, HER2-positivity increases [18F]FDG uptake while TNBC is associated with the highest [18F]FDG uptake of all clinical subtypes. Moreover, increased [18F]FDG uptake can be attributed to larger tumours in luminal and HER2-positive subtypes, but not in TNBC due to contradictory reports on its relative tumour size compared to other subtypes [93, 94]. This suggests underlying differences in [18F]FDG uptake mechanisms between clinical subtypes beyond receptor status, tumour size, proliferation and differentiation [95].

Distinct differences in [18F]FDG uptake between clinical subtypes could influence diagnostic, predictive or prognostic performance, especially when using cutoff values to predict outcome. To illustrate, applying the same cutoff value to different clinical subtypes to predict presence of axillary lymph node metastasis (ALNM) can lead to an underestimation of performance in TNBC since this subtype is associated with increased [18F]FDG uptake and a decreased rate of ALNM [40, 96]. Contrarily, Groheux et al. reported differences in baseline as well as percentage decrease [18F]FDG uptake in primary tumour response to NST between clinical subtypes, suggesting improved diagnostic performance when using distinct cutoffs [15]. In general, the precise effect of clinical subtypes on performance of [18F]FDG PET is lacking and the results of our meta-analysis suggest a need for more research on this topic.

While practices and guidelines differ, [18F]FDG PET/CT is generally recommended in breast cancer patients with a large primary tumour or with clinically node-positive disease [97]. While mainly performed to detect (distant) metastatic disease, the majority of primary tumours in breast cancer patients are [18F]FDG-avid [98]. In current clinical practice, [18F]FDG uptake is predominantly evaluated qualitatively. Considering the increasing number of studies reporting on the significant value of quantitative [18F]FDG PET, this imaging modality is not fully utilized by merely evaluating it qualitatively. Consequently, measuring [18F]FDG PET parameters such as SUV_max_ on the primary tumour could easily provide valuable predictive or prognostic information that could aid in clinical decision making in the context of personalized medicine. In addition, the application of artificial intelligence to [18F]FDG PET imaging provides a promising adjunct to further improve its diagnostic, predictive and prognostic accuracy [99].

The major limitations of this study were variability in the designs and methods of the included studies, specifically the variability in the administered dose of [18F]FDG, emission time, vendor, type of modality and cutoff values used for receptor status. This variability in design and methods (including vendor variability) is illustrated by the reported heterogeneities, hence the choice for SMD as a summary statistic. Including studies from 2007 onwards, differences in definitions with regard to receptor positivity as well as of criteria for clinical subtypes should be taken into account when interpreting the results of the meta-analyses in this study. Aware that varying definitions could influence the [18F]FDG uptake, there was deliberately chosen to incorporate these changes in the quality assessment instead of additional sensitivity analyses. Furthermore, it can be hypothesized that the changing criteria mainly relate to borderline cases that are of negligible effect on [18F]FDG uptake.

## Conclusions

This systematic review and meta-analysis indicates a substantial and significant association between increased [18F]FDG expressed as SUV_max_ and ER-negativity, PR-negativity, HER2-positivity and Ki-67-positivity. Clinical subtypes based on these markers follow the same pattern with lower [18F]FDG uptake in luminal subtypes including ER and PR, and higher uptake in TNBC. HER2 overexpression and associated subtypes have an intermediate effect on [18F]FDG uptake. Clinical subtypes should be taken into account when applying and interpreting [18F]FDG PET in breast cancer.

## Supplementary Information

**Additional file 1**. Full description of the methods with a delignation of the full-search algorithms for PubMed (Table S1) and Embase (Table S2).

**Additional file 2**. Overview of the characteristics of the included studies (Table S3), the [18F]FDG PET characteristics (Table S4), data extraction for the meta-analysis (Tables S5–S11) and methodologic quality of the included studies (Table S12).

## Data Availability

The datasets used and/or analysed during the current study are available from the corresponding author on reasonable request.

## Abbreviations

[18F]FDG: [18F]-fluorodeoxyglucose
ALNM: Axillary lymph node metastasis
CI: Confidence interval
CT: Computed tomography
ER: Estrogen receptor
GEP: Gene expression profiling
HER2: Human epidermal growth factor receptor 2
IHC: Immunohistochemical
LA: Luminal A
LB: Luminal B
MRI: Magnetic resonance imaging
NST: Neoadjuvant systemic therapy
PET: Positron emission tomography
PR: Progesterone receptor
QUADAS-2: Quality Assessment of Diagnostic Accuracy Studies 2
SD: Standard deviation
SMD: Standardized mean difference
SUV_max_: Maximum standardized uptake value
TNBC: Triple negative breast cancer

## References

[1] Allison KH, Hammond MEH, Dowsett M, McKernin SE, Carey LA, Fitzgibbons PL (2020). Estrogen and progesterone receptor testing in breast cancer: ASCO/CAP guideline update. J Clin Oncol.

[2] Cardoso F, Kyriakides S, Ohno S, Penault-Llorca F, Poortmans P, Rubio IT (2019). Early breast cancer: ESMO clinical practice guidelines for diagnosis, treatment and follow-up†. Ann Oncol.

[3] Wolff AC, Hammond MEH, Allison KH, Harvey BE, Mangu PB, Bartlett JMS (2018). Human epidermal growth factor receptor 2 testing in breast cancer: American Society of Clinical Oncology/College of American Pathologists clinical practice guideline focused update. J Clin Oncol.

[4] Sorlie T, Perou CM, Tibshirani R, Aas T, Geisler S, Johnsen H (2001). Gene expression patterns of breast carcinomas distinguish tumor subclasses with clinical implications. Proc Natl Acad Sci U S A.

[5] Perou CM, Sørlie T, Eisen MB, van de Rijn M, Jeffrey SS, Rees CA (2000). Molecular portraits of human breast tumours. Nature.

[6] Coates AS, Winer EP, Goldhirsch A, Gelber RD, Gnant M, Piccart-Gebhart M (2015). Tailoring therapies—improving the management of early breast cancer: St Gallen International Expert Consensus on the Primary Therapy of Early Breast Cancer 2015. Ann Oncol.

[7] Goldhirsch A, Winer EP, Coates AS, Gelber RD, Piccart-Gebhart M, Thürlimann B (2013). Personalizing the treatment of women with early breast cancer: highlights of the St Gallen International Expert Consensus on the Primary Therapy of Early Breast Cancer 2013. Ann Oncol.

[8] Goldhirsch A, Wood WC, Coates AS, Gelber RD, Thürlimann B, Senn HJ (2011). Strategies for subtypes--dealing with the diversity of breast cancer: highlights of the St. Gallen International Expert Consensus on the Primary Therapy of Early Breast Cancer 2011. Ann Oncol.

[9] Caresia Aroztegui AP, Garcia Vicente AM, Alvarez Ruiz S, Delgado Bolton RC, Orcajo Rincon J, Garcia Garzon JR (2017). 18F-FDG PET/CT in breast cancer: Evidence-based recommendations in initial staging. Tumour Biol.

[10] Diao W, Tian F, Jia Z (2018). The prognostic value of SUV(max) measuring on primary lesion and ALN by (18)F-FDG PET or PET/CT in patients with breast cancer. Eur J Radiol.

[11] Tian F, Shen G, Deng Y, Diao W, Jia Z (2017). The accuracy of (18)F-FDG PET/CT in predicting the pathological response to neoadjuvant chemotherapy in patients with breast cancer: a meta-analysis and systematic review. Eur Radiol.

[12] Gil-Rendo A, Martínez-Regueira F, Zornoza G, García-Velloso MJ, Beorlegui C, Rodriguez-Spiteri N (2009). Association between [18F]fluorodeoxyglucose uptake and prognostic parameters in breast cancer. BJS (Br J Surg).

[13] Groheux D, Giacchetti S, Moretti J-L, Porcher R, Espié M, Lehmann-Che J (2011). Correlation of high 18F-FDG uptake to clinical, pathological and biological prognostic factors in breast cancer. Eur J Nucl Med Mol Imaging.

[14] Kitajima K, Fukushima K, Miyoshi Y, Nishimukai A, Hirota S, Igarashi Y (2015). Association between ^18^F-FDG uptake and molecular subtype of breast cancer. Eur J Nucl Med Mol Imaging.

[15] Groheux D, Majdoub M, Sanna A, de Cremoux P, Hindié E, Giacchetti S (2015). Early metabolic response to neoadjuvant treatment: FDG PET/CT Criteria according to breast cancer subtype. Radiology.

[16] Koo HR, Park JS, Kang KW, Cho N, Chang JM, Bae MS (2014). 18F-FDG uptake in breast cancer correlates with immunohistochemically defined subtypes. Eur Radiol.

[17] AbdElaal AA, Zaher AM, Abdelgawad MI, Mekkawy MA, Eloteify LM (2021). Correlation of primary tumor metabolic parameters with clinical, histopathological and molecular characteristics in breast cancer patients at pre-operative staging FDG-PET/CT study. Egypt J Radiol Nucl Med.

[18] Abubakar Z, Reddy Akepati N, Bikkina P (2019). Correlation of maximum standardized uptake values in 18F-Fluorodeoxyglucose positron emission tomography-computed tomography scan with immunohistochemistry and other prognostic factors in breast cancer. Indian J Nucl Med.

[19] Ahn SG, Lee M, Jeon TJ, Han K, Lee HM, Lee SA (2014). [18F]-fluorodeoxyglucose positron emission tomography can contribute to discriminate patients with poor prognosis in hormone receptor-positive breast cancer. PLoS ONE.

[20] Akin M, Orguc S, Aras F, Kandiloglu AR (2020). Molecular subtypes of invasive breast cancer: correlation between PET/computed tomography and MRI findings. Nucl Med Commun.

[21] Arslan E, Aksoy T, Can Trabulus FD, Kelten Talu C, Yeni B, Çermik TF (2020). The association of 18F-fluorodeoxyglucose PET/computed tomography parameters with tissue gastrin-releasing peptide receptor and integrin αvβ3 receptor levels in patients with breast cancer. Nucl Med Commun.

[22] Arslan E, Çermik TF, Trabulus FDC, Talu ECK, Basaran S (2018). Role of 18F-FDG PET/CT in evaluating molecular subtypes and clinicopathological features of primary breast cancer. Nucl Med Commun.

[23] Baba S, Isoda T, Maruoka Y, Kitamura Y, Sasaki M, Yoshida T (2014). Diagnostic and Prognostic Value of Pretreatment SUV in ^18^F-FDG/PET in breast cancer: comparison with apparent diffusion coefficient from diffusion-weighted MR imaging. J Nucl Med.

[24] Basu S, Chen W, Tchou J, Mavi A, Cermik T, Czerniecki B (2008). Comparison of triple-negative and estrogen receptor-positive/progesterone receptor-positive/HER2-negative breast carcinoma using quantitative fluorine-18 fluorodeoxyglucose/positron emission tomography imaging parameters. Cancer.

[25] Bitencourt AGV, Lima ENP, Chojniak R, Marques EF, de Souza JA, Graziano L (2014). Correlation between PET/CT results and histological and immunohistochemical findings in breast carcinomas. Radiol Bras.

[26] Catalano OA, Horn GL, Signore A, Iannace C, Lepore M, Vangel M (2017). PET/MR in invasive ductal breast cancer: correlation between imaging markers and histological phenotype. Br J Cancer.

[27] Chang C-C, Tu H-P, Chen Y-W, Lin C-Y, Hou M-F (2014). Tumour and lymph node uptakes on dual-phased 2-deoxy-2-[18F]fluoro-D-glucose positron emission tomography/computed tomography correlate with prognostic parameters in breast cancer. J Int Med Res.

[28] Ekmekcioglu O, Aliyev A, Yilmaz S, Arslan E, Kaya R, Kocael P (2013). Correlation of 18F-fluorodeoxyglucose uptake with histopathological prognostic factors in breast carcinoma. Nucl Med Commun.

[29] García Vicente AM, Castrejón ÁS, Relea Calatayud F, Muñoz AP, León Martín AA, López-Muñiz IC (2012). 18F-FDG retention index and biologic prognostic parameters in breast cancer. Clin Nucl Med.

[30] Garcia Vicente AM, Soriano Castrejón A, Amo-Salas M, Lopez Fidalgo JF, Muñoz Sanchez MM, Alvarez Cabellos R (2016). Glycolytic activity in breast cancer using 18F-FDG PET/CT as prognostic predictor: a molecular phenotype approach. Rev Española Med Nucl Imagen Mol.

[31] Groheux D, Majdoub M, Tixier F, Le Rest CC, Martineau A, Merlet P (2015). Do clinical, histological or immunohistochemical primary tumour characteristics translate into different (18)F-FDG PET/CT volumetric and heterogeneity features in stage II/III breast cancer?. Eur J Nucl Med Mol Imaging.

[32] Humbert O, Berriolo-Riedinger A, Riedinger JM, Coudert B, Arnould L, Cochet A (2012). Changes in ^18^F-FDG tumor metabolism after a first course of neoadjuvant chemotherapy in breast cancer: influence of tumor subtypes. Ann Oncol.

[33] Humbert O, Riedinger JM, Chardin D, Desmoulins I, Brunotte F, Cochet A (2019). SUV calculation in breast cancer: which normalization should be applied when using 18F-FDG PET?. Q J Nucl Med Mol Imaging.

[34] Jeong Y-J, Jung J-W, Cho Y-Y, Park S-H, Oh H-K, Kang S (2017). Correlation of hypoxia inducible transcription factor in breast cancer and SUVmax of F-18 FDG PET/CT. Nucl Med Rev.

[35] Jo I, Zeon SK, Kim SH, Kim HW, Kang SH, Kwon SY (2015). Correlation of primary tumor FDG uptake with clinicopathologic prognostic factors in invasive ductal carcinoma of the breast. Nucl Med Mol Imaging.

[36] Jung NY, Kim SH, Choi BB, Kim SH, Sung MS (2015). Associations between the standardized uptake value of 18F-FDG PET/CT and the prognostic factors of invasive lobular carcinoma: in comparison with invasive ductal carcinoma. World J Surg Oncol.

[37] Kadoya T, Aogi K, Kiyoto S, Masumoto N, Sugawara Y, Okada M (2013). Role of maximum standardized uptake value in fluorodeoxyglucose positron emission tomography/computed tomography predicts malignancy grade and prognosis of operable breast cancer: a multi-institute study. Breast Cancer Res Treat.

[38] Keam B, Im S-A, Koh Y, Han S-W, Oh D-Y, Cho N (2011). Early metabolic response using FDG PET/CT and molecular phenotypes of breast cancer treated with neoadjuvant chemotherapy. BMC Cancer.

[39] Kim BS, Sung SH (2012). Usefulness of 18F-FDG uptake with clinicopathologic and immunohistochemical prognostic factors in breast cancer. Ann Nucl Med.

[40] Kim JY, Lee SH, Kim S, Kang T, Bae YT (2015). Tumour 18 F-FDG Uptake on preoperative PET/CT may predict axillary lymph node metastasis in ER-positive/HER2-negative and HER2-positive breast cancer subtypes. Eur Radiol.

[41] Kitajima K, Yamano T, Fukushima K, Miyoshi Y, Hirota S, Kawanaka Y (2016). Correlation of the SUVmax of FDG-PET and ADC values of diffusion-weighted MR imaging with pathologic prognostic factors in breast carcinoma. Eur J Radiol.

[42] Kwon HW, Lee JH, Pahk K, Park KH, Kim S (2021). Clustering subtypes of breast cancer by combining immunohistochemistry profiles and metabolism characteristics measured using FDG PET/CT. Cancer Imaging.

[43] Lee H, Lim HS, Ki SY, Park HM, Lee JE, Jeong WG (2021). 18F-fluorodeoxyglucose uptake on PET/computed tomography in association with androgen receptor expression and other clinicopathologic factors in surgically resected triple-negative breast cancer. Nucl Med Commun.

[44] Lee SS, Bae SK, Park YS, Park JS, Kim TH, Yoon HK (2017). Correlation of Molecular subtypes of invasive ductal carcinoma of breast with glucose metabolism in FDG PET/CT: based on the recommendations of the St. Gallen Consensus Meeting 2013. Nucl Med Mol Imaging.

[45] Liu J, Bian H, Zhang Y, Gao Y, Yin G, Wang Z (2021). Molecular subtype classification of breast cancer using established radiomic signature models based on 18F-FDG PET/CT images. Front Biosci Landmark.

[46] Miyake KK, Nakamoto Y, Kanao S, Tanaka S, Sugie T, Mikami Y (2014). JOURNAL CLUB: diagnostic value of 18F-FDG PET/CT and MRI in predicting the clinicopathologic subtypes of invasive breast cancer. Am J Roentgenol.

[47] Morawitz J, Kirchner J, Martin O, Bruckmann N-M, Dietzel F, Li Y (2021). Prospective correlation of prognostic immunohistochemical markers with SUV and ADC derived from dedicated hybrid breast 18F-FDG PET/MRI in women with newly diagnosed breast cancer. Clin Nucl Med.

[48] Nakajima N, Kataoka M, Sugawara Y, Ochi T, Kiyoto S, Ohsumi S (2013). Volume-based parameters of ^18^F-Fluorodeoxyglucose positron emission tomography/computed tomography improve disease recurrence prediction in postmastectomy breast cancer patients with 1 to 3 positive axillary lymph nodes. Int J Radiat Oncol Biol Phys.

[49] Noda Y, Goshima S, Kawada H, Kawai N, Koyasu H, Matsuo M. HER2-positive breast cancer: tumor-to-liver SUV ratio is the best parameter for detection in F-18 FDG-PET/CT. Iran J Radiol. 2017;14(3).

[50] Orsaria P, Chiaravalloti A, Caredda E, Marchese PV, Titka B, Anemona L (2018). Evaluation of the usefulness of FDG-PET/CT for nodal staging of breast cancer. Anticancer Res.

[51] Ozer N, Sahin A (2021). Correlation of breast cancer subgroups and axillary metastases with 18F-FDG PET/CT and the contribution of 18F-FDG PET/CT in the management of the axilla. J Coll Phys Surg Pak JCPSP.

[52] Payan N, Presles B, Brunotte F, Coutant C, Desmoulins I, Vrigneaud J-M (2020). Biological correlates of tumor perfusion and its heterogeneity in newly diagnosed breast cancer using dynamic first-pass 18F-FDG PET/CT. Eur J Nucl Med Mol Imaging.

[53] Qu YH, Long N, Ran C, Sun J (2021). The correlation of 18F-FDG PET/CT metabolic parameters, clinicopathological factors, and prognosis in breast cancer. Clin Transl Oncol.

[54] Ravina M, Saboury B, Chauhan MS, Jacob MJ, Pandit AG, Sanchety N (2019). Utility of (18) F-FDG PET/CT in pre-surgical risk stratification of patients with breast cancer. Hell J Nucl Med.

[55] Sengoz T, Karakaya YA, Gultekin A, Yaylali O, Senol H, Yuksel D (2022). Relationships of (18)F-FDG uptake by primary tumors with prognostic factors and molecular subtype in ductal breast cancer. Rev Esp Med Nucl Imagen Mol (Engl Ed).

[56] Song B-I, Hong CM, Lee HJ, Kang S, Jeong SY, Kim HW (2011). Prognostic value of primary tumor uptake on F-18 FDG PET/CT in patients with invasive ductal breast cancer. Nucl Med Mol Imaging.

[57] Straver ME, Aukema TS, Olmos RAV, Rutgers EJT, Gilhuijs KGA, Schot ME (2010). Feasibility of FDG PET/CT to monitor the response of axillary lymph node metastases to neoadjuvant chemotherapy in breast cancer patients. Eur J Nucl Med Mol Imaging.

[58] Tchou J, Sonnad SS, Bergey MR, Basu S, Tomaszewski J, Alavi A (2010). Degree of tumor FDG uptake correlates with proliferation index in triple negative breast cancer. Mol Imaging Biol.

[59] Ueda S, Tsuda H, Asakawa H, Shigekawa T, Fukatsu K, Kondo N (2008). Clinicopathological and prognostic relevance of uptake level using 18F-fluorodeoxyglucose positron emission tomography/computed tomography fusion imaging (18F-FDG PET/CT) in primary breast cancer. Jpn J Clin Oncol.

[60] Uğurluer G, Yavuz S, Çalıkuşu Z, Seyrek E, Kibar M, Serin M (2016). Correlation between 18F-FDG positron-emission tomography 18F-FDG uptake levels at diagnosis and histopathologic and immunohistochemical factors in patients with breast cancer. J Breast Health.

[61] Wu J, Wang S, Zhang X, Teng Z, Wang J, Yung BC (2018). (18)F-Alfatide II PET/CT for identification of breast cancer: a preliminary clinical study. J Nucl Med.

[62] Yildirim N, Simsek M, Aldemir MN, Bilici M, Tekin SB (2019). Relationship between 18-FDG-PET/CT and clinicopathological features and pathological responses in patients with locally advanced breast cancers. Eurasian J Med.

[63] Yoon H-J, Kang KW, Chun IK, Cho N, Im S-A, Jeong S (2014). Correlation of breast cancer subtypes, based on estrogen receptor, progesterone receptor, and HER2, with functional imaging parameters from 68Ga-RGD PET/CT and 18F-FDG PET/CT. Eur J Nucl Med Mol Imaging.

[64] Akdeniz N, Kömek H, Küçüköner M, Kaplan MA, Urakçi Z, Oruç Z (2021). The role of basal 18F-FDG PET/CT maximum standard uptake value and maximum standard uptake change in predicting pathological response in breast cancer patients receiving neoadjuvant chemotherapy. Nucl Med Commun.

[65] An Y-S, Kang DK, Jung YS, Han S, Kim TH (2015). Tumor metabolism and perfusion ratio assessed by 18F-FDG PET/CT and DCE-MRI in breast cancer patients: correlation with tumor subtype and histologic prognostic factors. Eur J Radiol.

[66] Can C, Komek H (2019). Metabolic and volume-based parameters of (18F)FDG PET/CT for primary mass and axillary lymph node metastasis in patients with invasive ductal carcinoma: a retrospective analysis in relation to molecular subtype, axillary lymph node metastasis and immunohistochemistry and inflammatory markers. Nucl Med Commun.

[67] Cerci SS, Bozkurt KK, Eroglu HE, Cerci C, Erdemoglu E, Bulbul PT (2016). Evaluation of the association between HIF-1α and HER-2 expression, hormone receptor status, Ki-67 expression, histology and tumor FDG uptake in breast cancer. Oncol Lett.

[68] Choi BB, Kim SH, Kang BJ, Lee JH, Song BJ, Jeong SH (2012). Diffusion-weighted imaging and FDG PET/CT: predicting the prognoses with apparent diffusion coefficient values and maximum standardized uptake values in patients with invasive ductal carcinoma. World J Surg Oncol.

[69] Cochet A, Pigeonnat S, Khoury B, Vrigneaud J-M, Touzery C, Berriolo-Riedinger A (2012). Evaluation of breast tumor blood flow with dynamic first-pass ^18^F-FDG PET/CT: comparison with angiogenesis markers and prognostic factors. J Nucl Med.

[70] Ege Aktas G, Tastekin E, Sarikaya A (2018). Assessment of biological and clinical aggressiveness of invasive ductal breast cancer using baseline 18F-FDG PET/CT-derived volumetric parameters. Nucl Med Commun.

[71] Has Simsek D, Sanli Y, Külle CB, Karanlik H, Kiliç B, Kuyumcu S (2017). Correlation of 18F-FDG PET/CT with pathological features and survival in primary breast cancer. Nucl Med Commun.

[72] Heudel P, Cimarelli S, Montella A, Bouteille C, Mognetti T (2010). Value of PET-FDG in primary breast cancer based on histopathological and immunohistochemical prognostic factors. Int J Clin Oncol.

[73] Higuchi T, Nishimukai A, Ozawa H, Fujimoto Y, Yanai A, Miyagawa Y (2016). Prognostic significance of preoperative ^18^F-FDG PET/CT for breast cancer subtypes. Breast.

[74] Iqbal R, Mammatas LH, Aras T, Vogel WV, van de Brug T, Oprea-Lager DE (2021). Diagnostic performance of [(18)F]FDG PET in staging grade 1–2, estrogen receptor positive breast cancer. Diagnostics (Basel).

[75] Ito M, Shien T, Kaji M, Mizoo T, Iwamoto T, Nogami T (2015). Correlation between 18F-fluorodeoxyglucose positron emission tomography/computed tomography and clinicopathological features in invasive ductal carcinoma of the breast. Acta Med Okayama.

[76] Kaida H, Toh U, Hayakawa M, Hattori S, Fujii T, Kurata S (2013). The relationship between 18F-FDG metabolic volumetric parameters and clinicopathological factors of breast cancer. Nucl Med Commun.

[77] Karan B, Pourbagher A, Torun N (2016). Diffusion-weighted imaging and 18F-fluorodeoxyglucose positron emission tomography/computed tomography in breast cancer: correlation of the apparent diffusion coefficient and maximum standardized uptake values with prognostic factors. J Magn Resonance Imaging.

[78] Koolen BB, Vrancken Peeters MJTFD, Wesseling J, Lips EH, Vogel WV, Aukema TS (2012). Association of primary tumour FDG uptake with clinical, histopathological and molecular characteristics in breast cancer patients scheduled for neoadjuvant chemotherapy. Eur J Nucl Med Mol Imaging.

[79] Masumoto N, Kadoya T, Sasada S, Emi A, Arihiro K, Okada M (2018). Intratumoral heterogeneity on dedicated breast positron emission tomography predicts malignancy grade of breast cancer. Breast Cancer Res Treat.

[80] Moon H, Noh WC, Kim H-A, Kim E-K, Park KW, Lee SS (2016). The relationship between estrogen receptor, progesterone receptor and human epidermal growth factor receptor 2 expression of breast cancer and the retention index in dual phase 18F-FDG PET/CT. Nucl Med Mol Imaging.

[81] Önner H, Coskun N, Erol M, Karanis MİE (2021). Asociación de características de textura de la PET/TC con [18F]FDG con las características inmunohistoquímicas en el cáncer de mama ductal infiltrante. Rev Española Med Nucl Imagen Mol.

[82] Ozen A, Altinay S, Ekmekcioglu O, Albayrak R, Muhammedoglu A, Yigitbas H (2016). Dual-time 18F-FDG PET/CT imaging in initial locoregional staging of breast carcinoma: comparison with conventional imaging and pathological prognostic factors. Indian J Surg.

[83] Sanli Y, Kuyumcu S, Ozkan ZG, Işık G, Karanlik H, Guzelbey B (2012). Increased FDG uptake in breast cancer is associated with prognostic factors. Ann Nucl Med.

[84] Sasada S, Masumoto N, Suzuki E, Sueoka S, Goda N, Kajitani K (2019). Prediction of biological characteristics of breast cancer using dual-phase FDG PET/CT. Eur J Nucl Med Mol Imaging.

[85] Sasaki M, Tozaki M, Kubota K, Murakami W, Yotsumoto D, Sagara Y (2018). Simultaneous whole-body and breast 18F-FDG PET/MRI examinations in patients with breast cancer: a comparison of apparent diffusion coefficients and maximum standardized uptake values. Jpn J Radiol.

[86] Tural D, Kivrak Salim D, Mutlu H, Erkilic M, Gunduz S, Karakurt M (2015). Is there any relation between PET-CT SUVmax value and prognostic factors in locally advanced breast cancer?. J Buon.

[87] Kim SJ, Kim S-J, Kim IJ, Pak K, Kim BS, Shin S (2016). Factors associated with 18F-Fluorodeoxyglucose uptake in T1 and T2 invasive ductal carcinoma of the breast. Nucl Med Mol Imaging.

[88] Inwald EC, Klinkhammer-Schalke M, Hofstädter F, Zeman F, Koller M, Gerstenhauer M (2013). Ki-67 is a prognostic parameter in breast cancer patients: results of a large population-based cohort of a cancer registry. Breast Cancer Res Treat.

[89] Oshiro C, Yamasaki M, Noda Y, Nishimae A, Takahashi H, Inaji H (2020). Comparative evaluation of nuclear and histological grades as prognostic factors for invasive breast cancer. Breast Cancer.

[90] Dunnwald LK, Rossing MA, Li CI (2007). Hormone receptor status, tumor characteristics, and prognosis: a prospective cohort of breast cancer patients. Breast Cancer Res BCR.

[91] Soret M, Bacharach SL, Buvat I (2007). Partial-volume effect in PET tumor imaging. J Nucl Med.

[92] Iorfida M, Maiorano E, Orvieto E, Maisonneuve P, Bottiglieri L, Rotmensz N (2012). Invasive lobular breast cancer: subtypes and outcome. Breast Cancer Res Treat.

[93] Wiechmann L, Sampson M, Stempel M, Jacks LM, Patil SM, King T (2009). Presenting features of breast cancer differ by molecular subtype. Ann Surg Oncol.

[94] Vasconcelos I, Hussainzada A, Berger S, Fietze E, Linke J, Siedentopf F (2016). The St. Gallen surrogate classification for breast cancer subtypes successfully predicts tumor presenting features, nodal involvement, recurrence patterns and disease free survival. Breast.

[95] Choi J, Kim DH, Jung WH, Koo JS (2013). Metabolic interaction between cancer cells and stromal cells according to breast cancer molecular subtype. Breast Cancer Res.

[96] Holm-Rasmussen EV, Jensen MB, Balslev E, Kroman N, Tvedskov TF (2015). Reduced risk of axillary lymphatic spread in triple-negative breast cancer. Breast Cancer Res Treat.

[97] Groheux D, Cochet A, Humbert O, Alberini JL, Hindie E, Mankoff D (2016). (1)(8)F-FDG PET/CT for staging and restaging of breast cancer. J Nucl Med.

[98] Groheux D, Giacchetti S, Moretti JL, Porcher R, Espie M, Lehmann-Che J (2011). Correlation of high 18F-FDG uptake to clinical, pathological and biological prognostic factors in breast cancer. Eur J Nucl Med Mol Imaging.

[99] Urso L, Manco L, Castello A, Evangelista L, Guidi G, Castellani M (2022). PET-derived radiomics and artificial intelligence in breast cancer: a systematic review. Int J Mol Sci.

