# Supplementary material for: The influence of receptor expression and clinical subtypes on baseline [18F]FDG uptake in breast cancer: systematic review and meta-analysis
Source: EJNMMI Res. 2023 Jan 23;13:5. doi: 10.1186/s13550-023-00953-y (PMC9871105; doi:10.1186/s13550-023-00953-y)
Supplement: Supplementary file 1 — Additional file 1. Full description of the methods with a delignation of the full-search algorithms for PubMed (Table S1) and Embase (Table S2). [file 13550_2023_953_MOESM1_ESM.docx]

**Manuscript information**

**Title:** The influence of receptor expression and clinical subtypes on baseline [18F]FDG uptake in breast cancer – systematic review and meta-analysis

**Journal:** EJNMMI Research

**First author:** Cornelis M. de Mooij

**Affiliation:** Maastricht University Medical Centre+, Maastricht, The Netherlands

**E-mail address:** [cmdemooij@hotmail.nl](mailto:cmdemooij@hotmail.nl)

**Methods**

***Literature Search***

This systematic review was conducted in accordance with the Preferred Reporting Items for Systematic Reviews and Meta-Analysis (PRISMA) statement (*1*). A comprehensive search was performed in the medical databases PubMed and Embase to identify eligible studies from inception until June 27, 2022. The full-search algorithms are delignated in Tables S1-S2. In addition, a manual cross-reference search of eligible papers was performed to identify additional studies eligible for inclusion.

***Study Selection***

After removal of duplicate studies two reviewers (C.M.d.M. and R.P.) independently screened the titles and abstracts to assess eligibility. Subsequently, full-text articles of all potentially eligible studies were read to decide if the study met all the predefined inclusion criteria. A consensus was reached by the two reviewers in case of discrepancies. Lastly, in case of multiple studies reporting on overlapping samples, the sample size per receptor was evaluated and for each receptor the study with the largest sample size was included in the meta-analysis. With regard to IHC-based clinical subtypes, the study with the largest total sample size, defined as the number of patients of all clinical subtypes combined, was included.

Studies were eligible for inclusion if they met the following criteria: patients with invasive breast cancer; [18F]FDG PET performed before any therapy; [18F]FDG uptake measured on the primary tumor and expressed as SUV_max_; IHC expression of ER, PR, HER2, or Ki-67 determined on specimens obtained by gross needle aspiration biopsy or surgical procedures; [18F]FDG uptake compared between patients negative and positive for IHC expression of ER, PR, HER2, or Ki-67; and [18F]FDG uptake compared between breast cancer clinical subtypes based on the IHC expression of ER, PR, HER2, or Ki-67 (e.g. St. Gallen consensus recommendations) (*2-4*). Studies only including patients with inflammatory breast cancer, duplicate studies on overlapping samples with a smaller sample size, studies written in a language other than English, reviews, conference abstracts, case reports, technical reports, and editorials were excluded. No unpublished data or data from abstracts was used.

***Data Extraction and quality assessment***

Data extraction and quality assessment was performed by two reviewers (C.M. and R.P.) independently and a consensus was reached with a third reviewer (T.N.) in the case of discrepancies. Data on the number of patients and mean and standard deviation (SD) of SUV_max_ of patients negative and positive for IHC expression of ER, PR, HER2, and Ki-67, as well as of clinical subtypes based on the IHC expression of ER, PR, HER2 or Ki-67, was extracted. Measures of spread reported as 95% confidence interval (CI) or standard error (SE) was converted to SD (*5*). If SUV_max_ was reported as median and (interquartile) range, these values were transformed to mean and SD (*6-8*). Additionally, demographic data (year and country of publication), study design characteristics (prospective or retrospective), histopathological data (distribution of histological type), [18F]FDG PET characteristics (modality, type of scanner, [18F]FDG dose, emission time and outlining method for tumor region of interest) were extracted.

Study quality was assessed by using the established Quality Assessment of Diagnostic Accuracy Studies 2 (QUADAS-2) tool (*9*). To ensure that QUADAS-2 is applicable in the present study, histopathology was set as the reference standard and [18F]FDG PET as the index test.

***Statistical Analysis***

Multiple meta-analyses were performed using data from (1) studies that compared negative with positive receptor status for ER, PR, HER2 or Ki-67 and (2) studies that compared clinical subtypes based on these markers. The primary summary statistic used in the meta-analyses was the standardized mean difference (SMD) with 95% CIs using Hedges’ g correction for small study samples. The SMD was preferred over the mean difference (MD) to take the influence of [18F]FDG dose, PET scanner type and settings and varying definitions for receptor positivity or subtypes into account. The SMD is the difference in mean values divided by the pooled SDs and expresses the mean difference between groups in units of SD. The SMD thus depends on both the size of the effect (i.e. the difference between means) and the SD (i.e. the inherent variability among patients). As we anticipated considerable between-study heterogeneity, a random-effects model (inverse variance method) was used to pool effect sizes. The DerSimonian-Laird estimator was used to calculate the heterogeneity variance τ^2^ (*10,11*). Knapp-Hartung adjustments were used to calculate the 95% confidence intervals around the pooled effect (*12*). The heterogeneity across studies was measured by using the statistic *I^2^*. This statistic quantifies the percentage of total variation across studies that is due to heterogeneity rather than chance (*13*). The primary analyses were based on the studies which presented mean [18F]FDG uptake with SD. Sensitivity analyses also included studies which presented median values with (interquartile) range. These values were transformed to mean values with SD and the impact of the addition of these studies on the results was evaluated. Egger’s regression test was used to identify small-study effects by testing for funnel plot asymmetry. The ‘meta’ and ‘metafor’ packages in R (R Foundation for Statistical Computing, version 4.1.2) were used for the statistical analyses (*14,15*).

***Table S1. PubMed Search***

| ("Breast Neoplasms"[MeSH Terms] OR (("Breast"[MeSH Terms] OR "breast*"[Title/Abstract] OR "mamma*"[Title/Abstract]) AND ("neoplas*"[Title/Abstract] OR "tumor"[Title/Abstract] OR "tumors"[Title/Abstract] OR "tumour"[Title/Abstract] OR "tumours"[Title/Abstract] OR "cancer*"[Title/Abstract] OR "malign*"[Title/Abstract] OR "carcinom*"[Title/Abstract]))) AND ("positron-Emission Tomography"[MeSH Terms] OR "positron emission tomograph*"[Title/Abstract] OR "PET"[Title/Abstract]) AND ("Fluorodeoxyglucose F18"[MeSH Terms] OR "FDG"[Title/Abstract] OR "fluorodeoxyglucose"[Title/Abstract] OR "fludeoxyglucose"[Title/Abstract] OR "SUV"[Title/Abstract] OR "standardized uptake value*"[Title/Abstract]) AND ("subtype*"[Title/Abstract] OR "immunohistochemistry"[MeSH Terms] OR "immunohistochemi*"[Title/Abstract] OR "IHC"[Title/Abstract] OR "luminal"[Title/Abstract] OR "triple negative breast neoplasms"[MeSH Terms] OR "triple negativ*"[Title/Abstract] OR "TNBC"[Title/Abstract] OR "basal"[Title/Abstract] OR "receptors, estrogen"[MeSH Terms] OR "estrogen"[Title/Abstract] OR "oestrogen"[Title/Abstract] OR "receptors, progesterone"[MeSH Terms] OR "progesteron*"[Title/Abstract] OR "receptor, erbb 2"[MeSH Terms] OR "c-erbB-2"[Title/Abstract] OR "CerbB2"[Title/Abstract] OR "human epidermal growth factor receptor"[Title/Abstract] OR "HER2"[Title/Abstract] OR "HER 2"[Title/Abstract] OR "Ki-67 Antigen"[MeSH Terms] OR "Ki67"[Title/Abstract] OR "Ki 67"[Title/Abstract] OR "MIB1"[Title/Abstract] OR "MIB 1"[Title/Abstract]) |
| --- |

***Table S2. Embase search***

| 1 | exp breast tumor/ | 29 | subtype*.ti,ab,kw. |
| --- | --- | --- | --- |
| 2 | exp breast/ | 30 | exp immunohistochemistry/ |
| 3 | breast*.ti,ab,kw. | 31 | immunohistochemi*.ti,ab,kw. |
| 4 | mamma*.ti,ab,kw. | 32 | IHC.ti,ab,kw. |
| 5 | 2 or 3 or 4 | 33 | exp triple negative breast cancer/ |
| 6 | exp malignant neoplasm/ | 34 | triple negativ*.ti,ab,kw. |
| 7 | neoplas*.ti,ab,kw. | 35 | TNBC.ti,ab,kw. |
| 8 | tumor.ti,ab,kw. | 36 | basal.ti,ab,kw. |
| 9 | tumors.ti,ab,kw. | 37 | luminal.ti,ab,kw. |
| 10 | tumour.ti,ab,kw. | 38 | exp estrogen receptor/ |
| 11 | tumours.ti,ab,kw. | 39 | estrogen.ti,ab,kw. |
| 12 | cancer*.ti,ab,kw. | 40 | oestrogen.ti,ab,kw. |
| 13 | malign*.ti,ab,kw. | 41 | exp progesterone receptor/ |
| 14 | carcinom*.ti,ab,kw. | 42 | progesteron*.ti,ab,kw. |
| 15 | 6 or 7 or 8 or 9 or 10 or 11 or 12 or 13 or 14 | 43 | exp human epidermal growth factor receptor 2 positive breast cancer/ |
| 16 | 5 and 15 | 44 | c-erbB-2.ti,ab,kw. |
| 17 | 1 or 16 | 45 | CerbB2.ti,ab,kw. |
| 18 | exp positron emission tomography/ | 46 | human epidermal growth factor receptor.ti,ab,kw. |
| 19 | positron emission tomograph*.ti,ab,kw. | 47 | HER2.ti,ab,kw. |
| 20 | PET.ti,ab,kw. | 48 | HER 2.ti,ab,kw. |
| 21 | 18 or 19 or 20 | 49 | exp Ki 67 antigen/ |
| 22 | exp fluorodeoxyglucose f 18/ | 50 | Ki67.ti,ab,kw. |
| 23 | FDG.ti,ab,kw. | 51 | Ki 67.ti,ab,kw. |
| 24 | fluorodeoxyglucose.ti,ab,kw. | 52 | MIB1.ti,ab,kw. |
| 25 | fludeoxyglucose.ti,ab,kw. | 53 | MIB 1.ti,ab,kw. |
| 26 | SUV.ti,ab,kw. | 54 | 29 or 30 or 31 or 32 or 33 or 34 or 35 or 36 or 37 or 38 or 39 or 40 or 41 or 42 or 43 or 44 or 45 or 46 or 47 or 48 or 49 or 50 or 51 or 52 or 53 |
| 27 | standardized uptake value*.ti,ab,kw. | 55 | 17 and 21 and 28 and 54 |
| 28 | 22 or 23 or 24 or 25 or 26 or 27 |  |  |

**References**

**1.** Moher D, Liberati A, Tetzlaff J, Altman DG. Preferred reporting items for systematic reviews and meta-analyses: the PRISMA statement. *BMJ.* 2009;339:b2535.

**2.** Coates AS, Winer EP, Goldhirsch A, et al. Tailoring therapies—improving the management of early breast cancer: St Gallen International Expert Consensus on the Primary Therapy of Early Breast Cancer 2015. *Annals of Oncology.* 2015;26:1533-1546.

**3.** Goldhirsch A, Winer EP, Coates AS, et al. Personalizing the treatment of women with early breast cancer: highlights of the St Gallen International Expert Consensus on the Primary Therapy of Early Breast Cancer 2013. *Annals of Oncology.* 2013;24:2206-2223.

**4.** Goldhirsch A, Wood WC, Coates AS, Gelber RD, Thürlimann B, Senn HJ. Strategies for subtypes--dealing with the diversity of breast cancer: highlights of the St. Gallen International Expert Consensus on the Primary Therapy of Early Breast Cancer 2011. *Ann Oncol.* 2011;22:1736-1747.

**5.** Higgins JPT TJ, Chandler J, Cumpston M, Li T, Page MJ, Welch VA (editors). Cochrane Handbook for Systematic Reviews of Interventions version 6.2 (updated February 2021). Cochrane, 2021. Available from [www.training.cochrane.org/handbook](file:///C:\Users\Kees%20de%20Mooij\surfdrive\PhD\26%20-%20Review%20subtypen\Manuscript\EJNMMI\www.training.cochrane.org\handbook).

**6.** Wan X, Wang W, Liu J, Tong T. Estimating the sample mean and standard deviation from the sample size, median, range and/or interquartile range. *BMC Med Res Methodol.* 2014;14:135.

**7.** Shi J, Luo D, Weng H, et al. Optimally estimating the sample standard deviation from the five-number summary. *Res Synth Methods.* 2020;11:641-654.

**8.** Luo D, Wan X, Liu J, Tong T. Optimally estimating the sample mean from the sample size, median, mid-range, and/or mid-quartile range. *Stat Methods Med Res.* 2018;27:1785-1805.

**9.** Whiting PF, Rutjes AWS, Westwood ME, et al. QUADAS-2: A Revised Tool for the Quality Assessment of Diagnostic Accuracy Studies. *Annals of Internal Medicine.* 2011;155:529-536.

**10.** DerSimonian R, Laird N. Meta-analysis in clinical trials. *Control Clin Trials.* 1986;7:177-188.

**11.** DerSimonian R, Laird N. Meta-analysis in clinical trials revisited. *Contemp Clin Trials.* 2015;45:139-145.

**12.** Knapp G, Hartung J. Improved tests for a random effects meta-regression with a single covariate. *Stat Med.* 2003;22:2693-2710.

**13.** Higgins JP, Thompson SG, Deeks JJ, Altman DG. Measuring inconsistency in meta-analyses. *Bmj.* 2003;327:557-560.

**14.** Balduzzi S, Rücker G, Schwarzer G. How to perform a meta-analysis with R: a practical tutorial. *Evid Based Ment Health.* 2019;22:153-160.

**15.** Viechtbauer W. Conducting Meta-Analyses in R with the metafor Package. *Journal of Statistical Software.* 2010;36:1 - 48.
