# Supplementary material for: The influence of receptor expression and clinical subtypes on baseline [18F]FDG uptake in breast cancer: systematic review and meta-analysis
Source: EJNMMI Res. 2023 Jan 23;13:5. doi: 10.1186/s13550-023-00953-y (PMC9871105; doi:10.1186/s13550-023-00953-y)
Supplement: Supplementary file 2 — Additional file 2. Overview of the characteristics of the included studies (Table S3), the [18F]FDG PET characteristics (Table S4), data extraction for the meta-analysis (Tables S5–S11) and methodologic quality of the included studies (Table S12). [file 13550_2023_953_MOESM2_ESM.docx]

**Manuscript information**

**Title:** The influence of receptor expression and clinical subtypes on baseline [18F]FDG uptake in breast cancer – systematic review and meta-analysis

**Journal:** EJNMMI Research

**First author:** Cornelis M. de Mooij

**Affiliation:** Maastricht University Medical Centre+, Maastricht, The Netherlands

**E-mail address:** [cmdemooij@hotmail.nl](mailto:cmdemooij@hotmail.nl)

## Additional file 2: Table S3. Characteristics of included studies

| Author | Year | Country | Design | Histology* | Meta-analysis |
| --- | --- | --- | --- | --- | --- |
| *Mean and SD* | | | | | |
| AbdElaal (*17*) | 2021 | Egypt | P | 36/3/1 | ER, PR, HER2, subtype^1^ |
| Abubakar (*18*) | 2019 | India | R | 197/2/20 | HER2, Ki-67, subtype^1^ |
| Ahn (*19*) | 2014 | Korea | R | nr | ER, PR, HER2, Ki-67, subtype^1^ |
| Akin (*20*) | 2020 | Turkey | R | nr | subtype^1^ |
| Arslan (*21*) | 2020 | Turkey | P | 67/1/22 | ER, PR, HER2, Ki-67, subtype^1^ |
| Arslan (*22*) | 2018 | Turkey | R | 416/29/57 | ER, PR, HER2, Ki-67, subtype^1^ |
| Baba (*23*) | 2014 | Japan | R | 65/0/5 | ER, PR, HER2, TNBC |
| Basu (*24*) | 2007 | USA | P | 58/7/9† | Subtype^2^ |
| Bitencourt (*25*) | 2014 | Brazil | P | 47/6/6 | Subtype^1^ |
| Catalano (*26*) | 2017 | USA | R | 49/0/0 | ER, PR, HER2, Ki-67 |
| Chang (*27*) | 2014 | Taiwan | R | 28/2/2 | ER, PR, HER2, TNBC |
| Ekmekcioglu (*28*) | 2013 | Turkey | nr | 104/9/23 | ER, PR, HER2, Ki-67, TNBC |
| García Vicente (*29*) | 2012 | Spain | P | 55/6/2† | ER, PR, HER2 |
| García Vicente (*30*) | 2016 | Spain | P | 175/19/0 | Subtype^1^ |
| Gil-Rendo (*12*) | 2009 | Spain | nr | 221/25/18 | ER, HER2, Ki-67 |
| Groheux (*31*) | 2015 | France | R | 156/7/8 | ER, PR, subtype^2^ |
| Humbert (*32*) | 2012 | France | P | 109/6/0 | PR, HER2 |
| Humbert (*33*) | 2019 | France | P | 232/17/3 | ER, subtype^2^ |
| Jeong (*34*) | 2017 | Korea | R | 207/0/0 | ER, PR, HER2 |
| Jo (*35*) | 2015 | Korea | R | 136/0/0 | ER, PR, HER2, TNBC |
| Jung (*36*) | 2015 | Korea | R | 73/32/0 | ER, PR, HER2, Ki-67 |
| Kadoya (*37*) | 2013 | Japan | P | nr | ER, PR, HER2 |
| Keam (*38*) | 2011 | Korea | P | 74/0/4 | ER, PR, HER2, Ki-67, TNBC, subtype^1^ |
| Kim (*39*) | 2012 | Korea | R | 91/0/0 | ER, PR, HER2, Ki-67, TNBC |
| Kim (*87*) | 2016 | Korea | R | 258/0/0 | ER, PR, HER2 |
| Kitajima (*14*) | 2015 | Japan | R | 282/13/13 | ER, PR, HER2, Ki-67, subtype^1^ |
| Kitajima (*41*) | 2016 | Japan | R | 201/8/7 | TNBC |
| Koo (*16*) | 2014 | Korea | R | 484/27/41 | ER, PR, HER2, Ki-67, subtype^1^ |
| Kwon (*42*) | 2021 | Korea | R | 242/16/31 | Subtype^1^ |
| Lee (*43*) | 2021 | Korea | R | 132/1/23 | Ki-67 |
| Lee (*44*) | 2017 | Korea | R | 183/0/0 | ER, PR, HER2, Ki-67, subtype^1^ |
| Liu (*45*) | 2021 | China | R | 263/3/7 | HER2, TNBC |
| Miyake (*46*) | 2014 | Japan | R | 79/4/6 | Subtype^1^ |
| Morawitz (*47*) | 2021 | Germany | P | 47/9/0 | Subtype^1^ |
| Nakajima (*48*) | 2013 | Japan | R | 93/0/0 | ER, PR, HER2, TNBC |
| Noda (*49*) | 2017 | Japan | R | nr | Subtype^1^ |
| Orsaria (*50*) | 2018 | Italy | P | 46/4/0 | ER, PR, HER2, Ki-67, TNBC, subtype^1^ |
| Ozer (*51*) | 2021 | Turkey | nr | 71/13/6 | Subtype^1^ |
| Payan (*52*) | 2020 | France | P | 203/12/2 | Subtype^2^ |
| Qu (*53*) | 2021 | China | R | 97/12/16 | ER, PR, HER2, Ki-67 |
| Ravina (*54*) | 2019 | India | P | 100/4/9 | ER, PR, HER2, TNBC |
| Sengoz (*55*) | 2021 | Turkey | R | 150/0/0 | ER, PR, HER2, Ki-67, subtype^1^ |
| Song (*56*) | 2011 | Korea | nr | 55/0/0 | ER, PR, HER2 |
| Straver (*57*) | 2010 | The Netherlands | nr | 31/3/4 | Subtype^2^ |
| Tchou (*58*) | 2010 | USA | R | 40/1/0 | TNBC |
| Ueda (*59*) | 2008 | Japan | nr | 136/9/7 | ER, PR, HER2 |
| Ugurluer (*60*) | 2016 | Turkey | R | 112/16/11 | ER, PR, HER2, TNBC |
| Wu (*61*) | 2018 | China | nr | nr | Subtype^1^ |

*Table S3. Continued.*

| Yildirim (*62*) | 2019 | Turkey | R | 46/3/2 | Ki-67, subtype^1^ |
| --- | --- | --- | --- | --- | --- |
| Yoon (*63*) | 2014 | Korea | P | 43/0/0 | ER, PR, HER2 |
| *Median and (interquartile) range* | | | | | |
| Akdeniz (*64*) | 2021 | Turkey | R | 172/9/31 | ER, PR, HER2, subtype^1^ |
| An (*65*) | 2015 | Korea | R | 67/0/0 | TNBC |
| Can (*66*) | 2019 | Turkey | R | 129/0/0 | Subtype^1^ |
| Cerci (*67*) | 2016 | Turkey | nr | nr | ER, PR, HER2, Ki-67 |
| Choi (*68*) | 2012 | Korea | nr | 118/0/0 | ER, PR, HER2, Ki-67 |
| Cochet (*69*) | 2012 | France | P | 38/2/0 | ER, PR, HER2, Ki-67, TNBC |
| Ege Aktas (*70*) | 2018 | Turkey | R | 65/0/0 | ER, PR, HER2, Ki-67, subtype^2^ |
| Groheux (*13*) | 2011 | France | P | 107/15/9 | HER2, TNBC |
| Has Simsek (*71*) | 2017 | Turkey | R | 357/22/57 | ER, PR, HER2, Ki-67, subtype^1^ |
| Heudel (*72*) | 2010 | France | P | 37/8/0 | ER, PR, HER2, TNBC |
| Higuchi (*73*) | 2016 | Japan | R | 350/11/26 | Subtype^2^ |
| Iqbal (*74*) | 2021 | The Netherlands | R | 57/17/1 | PR, HER2 |
| Ito (*75*) | 2015 | Japan | nr | 138/0/0 | ER, PR, HER2, Ki-67, subtype^1^ |
| Kaida (*76*) | 2013 | Japan | R | 84/0/8 | ER, PR, HER2, TNBC |
| Karan (*77*) | 2016 | Turkey | R | 56/3/11 | ER, PR, HER2 |
| Koolen (*78*) | 2012 | The Netherlands | P | 176/17/10 | ER, PR, HER2, Ki-67, subtype^2^ |
| Masumoto (*79*) | 2018 | Japan | R | nr | ER, HER2, Ki-67, subtype^1^ |
| Moon (*80*) | 2016 | Korea | R | 51/2/2 | ER, PR, HER2 |
| Önner (*81*) | 2021 | Turkey | R | 124/0/0 | Subtype^1^ |
| Ozen (*82*) | 2016 | Turkey | R | 67/2/10 | ER, PR |
| Sanli (*83*) | 2012 | Turkey | R | 68/2/9 | ER, PR, HER2, TNBC |
| Sasada (*84*) | 2019 | Japan | R | 439/21/40 | Subtype^1^ |
| Sasaki (*85*) | 2018 | Japan | R | 70/8/5 | ER, PR, HER2, Ki-67 |
| Tural (*86*) | 2015 | Turkey | R | 63/4/6 | Subtype^1^ |

*Abbreviations: *, Invasive NST or IDC/ILC/others; †, histological type not reported for all patients; nr, not reported; P, prospective; R, retrospective.*

## Additional file 2: Table S4. [18F]FDG PET scan characteristics and method of delineation

| Author | Modality | Dose (MBq) | Emission time (min) | Delineation | Scanner |
| --- | --- | --- | --- | --- | --- |
| *Mean and SD* | | | | | |
| AbdElaal (*17*) | PETCT | 3.7/kg | 2 | VOI | Biograph Horizon – Siemens |
| Abubakar (*18*) | PETCT | 5.18/kg | 3 | ROI | Discovery STE – GE |
| Ahn (*19*) | PET | 5.18/kg | nr | nr | Allegro – Philips |
| Akin (*20*) | PETCT | 5.4/kg | nr | ROI | TruFlight Select – Philips |
| Arslan (*21*) | PETCT | 3.7-5.2/kg | nr | ROI | Biograph mCT 20 – Siemens |
| Arslan (*22*) | PETCT | 3.7-5.2/kg | nr | ROI | (1) Biograph 6 – Siemens  (2) Biograph mCT 20 – Siemens |
| Baba (*23*) | PET | 3.7/kg | 2 | ROI | Advance Nxi – GE |
| Basu (*24*) | PET | nr | nr | ROI | Allegro – Philips |
| Bitencourt (*25*) | PETCT | 5.7/kg | nr | ROI | Gemini – Philips |
| Catalano (*26*) | PETMRI | 4.44/kg | 4 | Automated VOI | Biograph mMR – Siemens |
| Chang (*27*) | PETCT | 370 | 4 | ROI | Discovery ST16 – GE |
| Ekmekcioglu (*28*) | PETCT | 444-629 | 3 | ROI | Biograph 6 – Siemens |
| García Vicente (*29*) | PETCT | 370 | 3 | ROI | Discovery ST16 – GE |
| García Vicente (*30*) | PETCT | 370 | 3 | VOI | Discovery ST16 – GE |
| Gil-Rendo (*12*) | PET | 370 | nr | nr | ECAT EXACT – Siemens |
| Groheux (*31*) | PETCT | 5/kg | 2 | Automated VOI | Gemini XL – Philips |
| Humbert (*32*) | PETCT | 2-5/kg | nr | ROI | (1) C-PET Plus – Philips  (2) Gemini GXL – Philips |
| Humbert (*33*) | PETCT | 3-5/kg | 8 | VOI | (1) Gemini GXL – Philips  (2) Gemini TF – Philips |
| Jeong (*34*) | PETCT | 8.1/kg | 3 | ROI | Discovery STE – GE |
| Jo (*35*) | PETCT | 370 | nr | Circular ROI | (1) Discovery STE – GE  (2) Biograph mCT-64 – Siemens |
| Jung (*36*) | PETCT | 370-550 | 2 | ROI | (1) Biograph Classic – Siemens  (2) Biograph TruePoint – Siemens |
| Kadoya (*37*) | PETCT | 3.7/kg | 2-4 | ROI | (1) Discovery ST16 – GE  (2) Aquiduo – Toshiba |
| Keam (*38*) | PETCT | 5.18/kg | 2 | ROI | Gemini – Philips |
| Kim (*39*) | PET | 5.18/kg | 3 | Fixed ROI | Allegro – Philips |
| Kim (*87*) | PETCT | Nr | nr | VOI | (1) Gemini TF – Philips  (2) Biograph 40 – Siemens |
| Kitajima (*14*) | PETCT | 4/kg | 1.5 | ROI | Gemini GXL16 – Philips |
| Kitajima (*41*) | PETCT | 4/kg | 2 | VOI | Gemini GXL16 – Philips |
| Koo (*16*) | PETCT | 5.2/kg | 2-2.5 | ROI | (1) Biograph – Siemens  (2) Gemini – Philips |
| Kwon (*42*) | PETCT | 5.18/kg | 1 | ROI | Gemini TF – Philips |
| Lee (*43*) | PETCT | 5.55/kg | 2.5 | VOI | (1) Discovery 600 – GE  (2) Discovery ST – GE |
| Lee (*44*) | PETCT | 370 | 2 | VOI | Discovery STE – GE |
| Liu (*45*) | PETCT | 4/kg | 2 | Automated VOI | Discovery Elite – GE |
| Miyake (*46*) | PETCT | 3.7/kg | 2-3 | VOI | Discovery STE – GE |
| Morawitz (*47*) | PETMRI | 257±39 | 20 | ROI | Biograph mMR – Siemens |
| Nakajima (*48*) | PETCT | 3/kg | nr | VOI | Aquiduo – Toshiba |

*Table S4. Continued.*

| Noda (*49*) | PETCT | 2.7-5.9/kg | 2 | ROI | Biograph Sensation 16 – Siemens |
| --- | --- | --- | --- | --- | --- |
| Orsaria (*50*) | PETCT | 370-450 | 3.5 | ROI | Discovery Iq – Philips |
| Ozer (*51*) | PETCT | 369 | nr | nr | Ingenuity TF – Philips |
| Payan (*52*) | PETCT | 3/kg | 4 | VOI | Gemini TruFlight – Philips |
| Qu (*53*) | PETCT | 4.44-5.18/kg | 3 | ROI | Discovery 710 Clarity – GE |
| Ravina (*54*) | PETCT | 296-370 | 2 | ROI | Biograph 2 – Siemens |
| Sengoz (*55*) | PETCT | 259-407 | 1.5 | Automated VOI | Gemini TF – Philips |
| Song (*56*) | PETCT | 8.1/kg | 3 | ROI | (1) Reveal HiRez – Siemens  (2) Discovery STE – GE |
| Straver (*57*) | PETCT | 180-240 | 1.5 | nr | Gemini TF – Philips |
| Tchou (*58*) | PET | Nr | nr | ROI | Allegro – Philips |
| Ueda (*59*) | PETCT | 3.7/kg | 1.5 | ROI | Biograph Emotion – Siemens |
| Ugurluer (*60*) | PETCT | 5.55/kg | 3 | nr | Biograph TruePoint – Siemens |
| Wu (*61*) | PETCT | 3.7/kg | 3 | ROI | Biograph 16 – Siemens |
| Yildirim (*62*) | PETCT | 444 | nr | ROI | Biograph 16 – Siemens |
| Yoon (*63*) | PETCT | 5.18/kg | 2 | VOI | Biograph 40 – Siemens |
| *Median and (interquartile) range* | | | | | |
| Akdeniz (*64*) | PETCT | 3.5-5.5/kg | 2.5-3 | VOI | (1) Discovery Iq – Philips  (2) Biograph 6 – Siemens |
| An (*65*) | PETCT | 5/kg | 3 | VOI | Discovery ST – GE |
| Can (*66*) | PETCT | 5.5/kg | 3 | VOI | Biograph 6 – Siemens |
| Cerci (*67*) | PETCT | 3.7/kg | 2 | nr | Gemini TF – Philips |
| Choi (*68*) | PETCT | Nr | 3 | nr | (1) Biograph Duo – Siemens  (2) Biograph TruePoint – Siemens |
| Cochet (*69*) | PETCT | 5/kg | 8 | ROI | Gemini XL – Philips |
| Ege Aktas (*70*) | PETCT | 4.44/kg | nr | Semiautomatic VOI | Discovery STE8 – GE |
| Groheux (*13*) | PETCT | 5/kg | 2 | Circular ROI | Gemini XL – Philips |
| Has Simsek (*71*) | PETCT | 350-450 | 3 | ROI | Biograph TruePoint – Siemens |
| Heudel (*72*) | PETCT | 5/kg | nr | nr | Gemini – Philips |
| Higuchi (*73*) | PETCT | 4/kg | nr | ROI | Gemini GXL16 – Philips |
| Ito (*75*) | PETCT | 3.7/kg | nr | ROI | Biograph Sensation 16 – Siemens |
| Iqbal (*74*) | PETCT | 3.5/kg  190-240 | 2 | Semiautomatic VOI | (1) Gemini TF – Philips (1) Ingenuity TF – Philips  (2) Gemini TF – Philips  (4) Gemini TF Big Bore – Philips |
| Kaida (*76*) | PET | 4.44/kg | 2.5 | VOI | Allegro – Philips |
| Karan (*77*) | PETCT | 296-370 | 2.5 | ROI | Discovery STE8 – GE |
| Koolen (*78*) | PETCT | 180-240 | 3 | Automatic ROI | Gemini TF – Philips |
| Masumoto (*79*) | PETCT | 3.0-3.7/kg | 2-3 | ROI | Discovery ST16 – GE |
| Moon (*80*) | PETCT | 7.4/kg | 3.5 | Ellipsoid VOI | Biograph 6 – Siemens |
| Önner (*81*) | PETCT | 3.7/kg | 2 | ROI | Biograph Sensation 16 – Siemens |
| Ozen (*82*) | PETCT | 2.5/kg | 3 | ROI | Gemini XL – Philips |
| Sanli (*83*) | PETCT | 370-550 | 3 | nr | nr |
| Sasada (*84*) | PETCT | 3.0-3.7/kg | 2-3 | ROI | Discovery ST16 – GE |
| Sasaki (*85*) | PETMRI | 3.7/kg | 4 | VOI | Biograph mMR – Siemens |

*Table S4. Continued.*

| Tural (*86*) | PETCT | 260-370 | 3 | nr | Biograph 6 – Siemens |
| --- | --- | --- | --- | --- | --- |

*Abbreviations: nr, not reported; ROI, region of interest; VOI, volume of interest.*

## Additional file 2: Table S5. [18F]FDG uptake of ER-status

|  | **ER-positive** | | **ER-negative** | |
| --- | --- | --- | --- | --- |
| **Study** | **n** | **values** | **n** | **Values** |
| *Mean and SD* | | | | |
| AbdElaal (*17*) | 31 | 9.12 ± 5.70 | 9 | 16.7 ± 9.60 |
| Ahn (*19*) | 227 | 3.69 ± 2.43 | 28 | 3.74 ± 2.33 |
| Arslan (*21*) | 41 | 11.9 ± 7.2 | 49 | 16.6 ± 12.6 |
| Arslan (*22*) | 391 | 10.7 ± 7.1 | 111 | 15.8 ± 10.5 |
| Baba (*23*) | 37 | 6.19 ± 5.10 | 20 | 8.78 ± 5.89 |
| Catalano (*26*) | 14 | 6.18 ± 4.34 | 7 | 14.19 ± 7.17 |
| Chang (*27*) | 22 | 4.08 ± 2.82 | 10 | 5.79 ± 3.35 |
| Ekmekcioglu (*28*) | 98 | 9.64 ± 7.35 | 27 | 12.53 ± 5.34 |
| Garcia Vicente (*29*) | 41 | 6.8 ± 6.1 | 19 | 9.7 ± 6.4 |
| Gil-Rendo (*12*) | 175 | 3.36 ± 2.55 | 70 | 5.16 ± 3.56 |
| Groheux (*31*) | 95 | 6.4 ± 3.8 | 76 | 9.0 ± 5.9 |
| Humbert (*33*) | 158 | 7.9 ± 5.1 | 94 | 12.1 ± 7.6 |
| Jeong (*34*) | 144 | 3.4 ± 2.8 | 63 | 6.8 ± 4.9 |
| Jo (*35*) | 81 | 6.0 ± 6.0 | 55 | 11.6 ± 5.9 |
| Jung (*36*) | 84 | 2.77 ± 2.83 | 20 | 5.84 ± 5.10 |
| Kadoya (*37*) | 292 | 3.43 ± 2.77 | 52 | 5.01 ± 4.40 |
| Keam (*38*) | 38 | 6.4 ± 3.3 | 40 | 8.6 ± 4.9 |
| Kim (*39*) | 61 | 3.8 ± 2.9 | 30 | 4.8 ± 2.6 |
| Kim (*87*) | 176 | 5.53 ± 3.67 | 82 | 9.79 ± 6.53 |
| Kitajima (*14*) | 227 | 4.73 ± 3.30 | 81 | 7.00 ± 4.73 |
| Koo (*16*) | 398 | 4.99 ± 3.65 | 154 | 8.84 ± 5.64 |
| Lee (*44*) | 121 | 6.3 ± 4.2 | 62 | 9.4 ± 6.1 |
| Nakajima (*48*) | 73 | 5.3 ± 3.4 | 20 | 9.8 ± 8.5 |
| Orsaria (*50*) | 42 | 4.2 ± 2.8 | 8 | 5.1 ± 3.5 |
| Qu (*53*) | 89 | 5.72 ± 2.13 | 36 | 9.26 ± 3.27 |
| Ravina (*54*) | 38 | 6.74 ± 4.07 | 72 | 9.59 ± 6.19 |
| Sengoz (*55*) | 117 | 6.17 ± 3.58 | 28 | 6.98 ± 3.31 |
| Song (*56*) | 38 | 4.5 ± 3.4 | 17 | 10.3 ± 5.0 |
| Ueda (*59*) | 33 | 4.0 ± 3.0 | 119 | 6.4 ± 4.1 |
| Ugurluer (*60*) | 106 | 5.40 ± 4.12 | 33 | 8.70 ± 4.02 |
| Yoon (*63*) | 22 | 10.48 ± 6.01 | 21 | 15.88 ± 8.73 |
| *Median and (interquartile) range* | | | | |
| Akdeniz (*64*) | 143 | 6.5 (1.1 – 36.6)* | 69 | 10.6 (1.9 – 4.8)* |
| Cerci (*67*) | 76 | 6 (6.2)† | 16 | 9.6 (4.0)† |
| Choi (*68*) | 89 | 2.8 (0 – 20.5)* | 28 | 5.3 (0 – 18.7)* |
| Cochet (*69*) | 26 | 6.1 (4.6 – 8.9)‡ | 14 | 10.3 (6.8 – 12.9)‡ |
| Ege Aktas (*70*) | 43 | 8.5 (5.7 – 11.5)‡ | 22 | 10.2 (7.5 – 13.6)‡ |
| Has Simsek (*71*) | 308 | 9.3 (7.1 – 13.2)‡ | 117 | 12.3 (8.3 – 18.1)‡ |
| Heudel (*72*) | 27 | 2.4 (2.4 – 19.5)* | 18 | 7 (0.7 – 11.7)* |
| Ito (*75*) | 113 | 3.2 (2.3 – 5.5)‡ | 25 | 8.7 (4.0 – 13.2)‡ |
| Kaida (*76*) | 69 | 2.70 (1.10 – 9.10)* | 24 | 3.95 (1.30 – 11.80)* |
| Karan (*77*) | 57 | 6.41 (1.90 – 21.40)* | 13 | 14.00 (3.70 – 21.10)* |
| Koolen (*78*) | 132 | 5.7 (4.9)† | 82 | 9.1 (9.1)† |
| Masumoto (*79*) | 170 | 2.5 (1.4 – 3.9)‡ | 25 | 4.5 (2.6 – 6.5)‡ |
| Moon (*80*) | 30 | 7.2 (2.3 – 17.0)*  (3.5 – 11.1)‡ | 25 | 9.0 (3.2 – 17.6)*  (6.5 – 12.3)‡ |
| Ozen (*82*) | 63 | 4.26 (2.59 – 8.13)‡ | 15 | 8.72 (3.79 – 14.28)‡ |
| Sanli (*83*) | 56 | 11.25 (2.00 – 39.00)* | 23 | 15.00 (2.09 – 39.00)* |

*Table S5. Continued.*

| Sasaki (*85*) | 77 | 3.69 (0.60 – 23.49)* | 6 | 3.47 (2.48 – 4.67)* |
| --- | --- | --- | --- | --- |

*Abbreviations: SD, standard deviation; *, range; †, IQR (interquartile range); ‡, Q1-Q3 (quartile 1 and 3, respectively)*

## Additional file 2: Table S6. [18F]FDG uptake of PR-status

|  | **PR-positive** | | **PR-negative** | |
| --- | --- | --- | --- | --- |
| **Study** | **n** | **values** | **n** | **Values** |
| *Mean and SD* | | | | |
| AbdElaal (*17*) | 24 | 9.13 ± 6.20 | 16 | 13.37 ± 9.70 |
| Ahn (*19*) | 226 | 3.74 ± 2.47 | 39 | 3.43 ± 2.00 |
| Arslan (*21*) | 42 | 11.7 ± 7.2 | 48 | 16.9 ± 12.6 |
| Arslan (*22*) | 379 | 10.6 ± 7.1 | 123 | 15.5 ± 10.1 |
| Baba (*23*) | 28 | 5.40 ± 4.06 | 29 | 7.24 ± 5.83 |
| Catalano (*26*) | 12 | 6.18 ± 4.34 | 9 | 14.19 ± 7.17 |
| Chang (*27*) | 18 | 4.25 ± 3.04 | 14 | 5.04 ± 3.09 |
| Ekmekcioglu (*28*) | 65 | 10.32 ± 8.40 | 60 | 10.20 ± 5.29 |
| Garcia Vicente (*29*) | 26 | 6.1 ± 6.0 | 34 | 9.0 ± 6.4 |
| Groheux (*31*) | 57 | 5.8 ± 3.9 | 112 | 7.7 ± 5.4 |
| Humbert (*32*) | 60 | 5.3 ± 3.8 | 55 | 8.1 ± 7.2 |
| Jeong (*34*) | 151 | 3.8 ± 3.1 | 56 | 6.2 ± 5.1 |
| Jo (*35*) | 65 | 6.3 ± 5.7 | 71 | 10.2 ± 6.0 |
| Jung (*36*) | 70 | 2.71 ± 2.41 | 33 | 4.82 ± 5.00 |
| Kadoya (*37*) | 256 | 3.33 ± 2.67 | 88 | 4.68 ± 4.00 |
| Keam (*38*) | 30 | 6.5 ± 3.3 | 48 | 8.2 ± 4.8 |
| Kim (*39*) | 54 | 3.6 ± 2.5 | 37 | 5.0 ± 3.1 |
| Kim (*87*) | 162 | 5.72 ± 4.78 | 96 | 8.85 ± 5.20 |
| Kitajima (*14*) | 160 | 4.48 ± 3.03 | 148 | 6.24 ± 4.00 |
| Koo (*16*) | 310 | 4.93 ± 3.77 | 242 | 7.52 ± 5.19 |
| Lee (*44*) | 103 | 6.1 ± 4.2 | 80 | 8.9 ± 5.8 |
| Nakajima (*48*) | 54 | 5.5 ± 3.3 | 39 | 7.3 ± 7.0 |
| Orsaria (*50*) | 29 | 3.7 ± 2.5 | 21 | 5.3 ± 3.1 |
| Qu (*53*) | 66 | 4.01 ± 1.85 | 59 | 9.80 ± 3.12 |
| Ravina (*54*) | 35 | 6.23 ± 3.85 | 75 | 9.71 ± 6.15 |
| Sengoz (*55*) | 113 | 5.95 ± 2.96 | 32 | 7.68 ± 4.90 |
| Song (*56*) | 37 | 4.3 ± 2.7 | 18 | 10.4 ± 5.4 |
| Ueda (*59*) | 50 | 4.1 ± 2.9 | 102 | 5.4 ± 4.2 |
| Ugurluer (*60*) | 97 | 5.40 ± 3.94 | 42 | 8.00 ± 3.89 |
| Yoon (*63*) | 9 | 8.60 ± 5.64 | 34 | 14.05 ± 8.17 |
| *Median and (interquartile) range* | | | | |
| Akdeniz (*64*) | 136 | 6.6 (1.1 – 36.6)* | 76 | 11 (2.7 – 48.0)* |
| Cerci (*67*) | 75 | 6.0 (6.1)† | 17 | 9.8 (3.3)† |
| Choi (*68*) | 74 | 2.8 (0 – 14.1)* | 43 | 4.6 (0 – 20.5)* |
| Cochet (*69*) | 21 | 5.5 (3.1 – 9.3)‡ | 19 | 9.2 (6.5 – 12.7)‡ |
| Ege Aktas (*70*) | 36 | 7.1 (4.8 – 10.7)‡ | 29 | 9.9 (7.9 – 13.4)‡ |
| Has Simsek (*71*) | 234 | 9.1 (6.5 – 13.4)‡ | 185 | 11.5 (7.1 – 16.9)‡ |
| Heudel (*72*) | 17 | 2.2 (0.7 – 7.8)* | 28 | 6.0 (0.9 – 19.5)* |
| Iqbal (*74*) | 61 | 4.42 (3.13 – 6.42)‡ | 13 | 5.07 (4.08 – 7.67)‡ |
| Ito (*75*) | 95 | 3.1 (2.3 – 5.4)‡ | 43 | 6.3 (3.2 – 10.7)‡ |
| Kaida (*76*) | 46 | 2.6 (1.1 – 9.1)* | 47 | 3.5 (1.3 – 11.8)* |
| Karan (*77*) | 50 | 6.34 (1.90 – 21.40)* | 20 | 7.90 (2.28 – 21.10)* |
| Koolen (*78*) | 101 | 5.4 (4.3)† | 113 | 8.8 (8.1)† |
| Moon (*80*) | 43 | 7.9 (2.3 – 17.6)*  (5.3 – 12.8)‡ | 12 | 8.2 (3.5 – 11.6)*  (6.4 – 9.4)‡ |
| Ozen (*82*) | 55 | 4.15 (2.34 – 8.25)‡ | 23 | 6.37 (3.62 – 14.28)‡ |
| Sanli (*83*) | 43 | 10.00 (2.00 – 39.00)* | 36 | 11.84 (2.00 -39.00)* |

*Table S6. Continued.*

| Sasaki (*85*) | 66 | 3.84 (0.60 – 23.49)* | 17 | 3.01 (0.72 – 7.66)* |
| --- | --- | --- | --- | --- |

*Abbreviations: SD, standard deviation; *, range; †, IQR (interquartile range); ‡, Q1-Q3 (quartile 1 and 3, respectively)*

## Additional file 2: Table S7. [18F]FDG uptake of HER2-status

|  | **HER2-positive** | | **HER2-negative** | |
| --- | --- | --- | --- | --- |
| **Study** | **n** | **values** | **n** | **Values** |
| *Mean and SD* | | | | |
| AbdElaal (*17*) | 14 | 12.35 ± 5.80 | 26 | 10.01 ± 8.90 |
| Abubakar (*18*) | 149 | 11.49 ± 6.50 | 114 | 11.08 ± 5.05 |
| Ahn (*19*) | 47 | 5.12 ± 2.40 | 258 | 3.44 ± 2.33 |
| Arslan (*21*) | 34 | 14.1 ± 8.8 | 56 | 13.9 ± 12.2 |
| Arslan (*22*) | 119 | 13.6 ± 7.4 | 383 | 11.2 ± 8.4 |
| Baba (*23*) | 9 | 6.64 ± 3.26 | 48 | 7.45 ± 5.90 |
| Catalano (*26*) | 10 | 6.17 ± 4.02 | 11 | 11.79 ± 7.65 |
| Chang (*27*) | 9 | 5.35 ± 3.07 | 23 | 4.31 ± 3.05 |
| Ekmekcioglu (*28*) | 18 | 11.66 ± 6.39 | 106 | 9.87 ± 7.00 |
| Garcia Vicente (*29*) | 22 | 8.1 ± 6.5 | 38 | 7.5 ± 6.3 |
| Gil-Rendo (*12*) | 83 | 5.03 ± 4.53 | 167 | 3.37 ± 2.65 |
| Humbert (*32*) | 37 | 6.3 ± 4.5 | 78 | 6.8 ± 6.4 |
| Jeong (*34*) | 165 | 4.1 ± 3.4 | 42 | 5.6 ± 5.2 |
| Jo (*35*) | 57 | 9.2 ± 5.0 | 79 | 7.6 ± 5.7 |
| Jung (*36*) | 17 | 4.59 ± 4.16 | 74 | 3.13 ± 3.51 |
| Kadoya (*37*) | 47 | 5.01 ± 3.56 | 297 | 3.46 ± 2.99 |
| Keam (*38*) | 17 | 6.8 ± 3.2 | 61 | 7.7 ± 4.6 |
| Kim (*39*) | 17 | 5.1 ± 3.6 | 74 | 4.0 ± 2.6 |
| Kim (*87*) | 90 | 7.76 ± 6.10 | 168 | 6.41 ± 4.52 |
| Kitajima (*14*) | 57 | 7.02 ± 3.81 | 251 | 4.94 ± 3.49 |
| Koo (*16*) | 99 | 7.30 ± 4.72 | 453 | 5.80 ± 4.57 |
| Lee (*44*) | 51 | 8.9 ± 5.2 | 132 | 6.7 ± 5.0 |
| Liu (*45*) | 106 | 10.65 ± 5.67 | 167 | 9.94 ± 6.31 |
| Nakajima (*48*) | 19 | 7.5 ± 3.6 | 74 | 5.9 ± 5.5 |
| Orsaria (*50*) | 9 | 4.7 ± 3.0 | 41 | 4.3 ± 2.9 |
| Qu (*53*) | 37 | 11.56 ± 3.42 | 88 | 4.71 ± 1.76 |
| Ravina (*54*) | 47 | 8.87 ± 5.00 | 63 | 8.42 ± 6.19 |
| Sengoz (*55*) | 48 | 6.70 ± 3.44 | 98 | 6.19 ± 3.57 |
| Song (*56*) | 18 | 7.8 ± 5.9 | 37 | 5.6 ± 4.0 |
| Ueda (*59*) | 31 | 6.2 ± 4.2 | 114 | 4.0 ± 3.1 |
| Ugurluer (*60*) | 47 | 6.80 ± 4.11 | 92 | 5.90 ± 3.84 |
| Yoon (*63*) | 13 | 10.85 ± 5.14 | 30 | 14.10 ± 8.68 |
| *Median and (interquartile) range* | | | | |
| Akdeniz (*64*) | 88 | 8.9 (1.1 – 37.8)* | 124 | 6.8 (1.9 – 48.0)* |
| Cerci (*67*) | 30 | 7.9 (3.8)† | 62 | 5.7 (6.6)† |
| Choi (*68*) | 50 | 3.7 (0 – 20.5)* | 67 | 2.9 (0 – 16.1)* |
| Cochet (*69*) | 15 | 6.5 (5.5 – 8.1)‡ | 25 | 9.3 (4.5 – 13.0)‡ |
| Ege Aktas (*70*) | 25 | 11.2 (8.0 – 13.3)‡ | 40 | 8.0 (5.4 – 10.5)‡ |
| Groheux (*13*) | 24 | 6.7 (5.1 – 9.7)‡ | 106 | 6.2 (4.2 – 9.7)‡ |
| Has Simsek (*71*) | 74 | 12.0 (8.2 – 17.0)‡ | 349 | 10.0 (5.9 – 15.0)‡ |
| Heudel (*72*) | 9 | 3.5 (1.2 – 10.1)* | 36 | 3.7 (0.7 – 19.5)* |
| Iqbal (*74*) | 9 | 5.74 (4.53 – 9.99)‡ | 65 | 4.23 (3.13 – 6.37)‡ |
| Ito (*75*) | 20 | 5.5 (3.4 – 9.3)‡ | 116 | 3.5 (2.3 – 6.6)‡ |
| Kaida (*76*) | 42 | 2.9 (1.3 – 9.1)* | 51 | 2.8 (1.1 – 11.8)* |
| Karan (*77*) | 33 | 9.30 (2.28 – 21.40)* | 37 | 6.20 (1.90 – 17.70)* |
| Koolen (*78*) | 53 | 6.2 (4.8)† | 161 | 7.1 (6.5)† |
| Masumoto (*79*) | 28 | 4.2 (1.7 – 6.4)‡ | 167 | 2.5 (1.5 – 4.0)‡ |
| Moon (*80*) | 21 | 9.5 (2.3 – 16.3)*  (6.4 – 11.9)‡ | 34 | 7.2 (2.5 – 17.6)*  (5.1 – 11.1)‡ |

*Table S7. Continued.*

| Sanli (*83*) | 28 | 16.0 (5.0 – 39.0)* | 51 | 10.0 (2.0 – 39.0)* |
| --- | --- | --- | --- | --- |
| Sasaki (*85*) | 8 | 5.66 (1.26 – 10.06)* | 76 | 3.37 (0.60 – 23.49)* |

*Abbreviations: SD, standard deviation; *, range; †, IQR (interquartile range); ‡, Q1-Q3 (quartile 1 and 3, respectively)*

## Additional file 2: Table S8. [18F]FDG uptake Ki-67-status

|  | **Ki-67-positive** | | **Ki-67-negative** | |
| --- | --- | --- | --- | --- |
| **Study** | **n** | **values** | **n** | **Values** |
| *Mean and SD* | | | | |
| Abubakar (*18*) | 167 | 11.97±5.80 | 30 | 7.25 ± 3.40 |
| Ahn (*19*) | 33 | 4.70 ± 2.76 | 272 | 3.57 ± 2.34 |
| Arslan (*21*) | 87 | 14.6 ± 10.9 | 3 | 9.9 ± 2.0 |
| Arslan (*22*) | 445 | 12.4 ± 8.4 | 57 | 7.2 ± 4.1 |
| Catalano (*26*) | 9 | 10.31 ± 6.88 | 12 | 5.90 ± 4.97 |
| Ekmekcioglu (*28*) | 40 | 10.53 ± 5.69 | 24 | 5.82 ± 6.94 |
| Gil-Rendo (*12*) | 117 | 5.02 ± 4.86 | 127 | 2.86 ± 2.16 |
| Jung (*36*) | 42 | 5.41 ± 4.40 | 57 | 1.90 ± 1.87 |
| Keam (*38*) | 41 | 8.5 ± 4.4 | 30 | 6.2 ± 4.1 |
| Kim (*39*) | 57 | 4.9 ± 3.0 | 34 | 3.0 ± 2.1 |
| Kitajima (*14*) | 205 | 6.14 ± 3.84 | 103 | 3.70 ± 2.50 |
| Koo (*16*) | 105 | 8.87 ± 5.67 | 447 | 5.41 ± 4.08 |
| Lee (*43*) | 150 | 8.50 ± 6.82 | 6 | 3.1 ± 4.0 |
| Lee (*44*) | 121 | 8.7 ± 5.5 | 62 | 4.6 ± 2.9 |
| Orsaria (*50*) | 28 | 5.2 ± 3.3 | 22 | 3.3 ± 2.0 |
| Qu (*53*) | 52 | 9.18 ± 2.96 | 73 | 4.58 ± 1.67 |
| Sengoz (*55*) | 107 | 6.77 ± 3.44 | 31 | 4.48 ± 2.49 |
| Yildirim (*62*) | 34 | 7.18 ± 4.36 | 5 | 7.03 ± 6.16 |
| *Median and (interquartile) range* | | | | |
| Cerci (*67*) | 58 | 7.8 (5.5)† | 34 | 4.7 (6.8)† |
| Choi (*68*) | 67 | 4.8 (0 – 20.5)* | 50 | 2.3 (0 – 8.6)* |
| Cochet (*69*) | 17 | 12.1 (7.9 – 14.6)‡ | 23 | 5.7 (3.5 – 7.0)‡ |
| Ege Aktas (*70*) | 49 | 10.0 (7.3 – 13.3)‡ | 16 | 7.1 (4.3 – 8.4)‡ |
| Has Simsek (*71*) | 277 | 11.0 (7.0 – 17.6)‡ | 89 | 8.0 (4.7 – 13.0)‡ |
| Ito (*75*) | 71 | 5.5 (2.8 – 9.3)‡ | 67 | 2.9 (2.1 – 4.5)‡ |
| Koolen (*78*) | 133 | 8.2 (6.6)† | 80 | 5.4 (5.4)† |
| Masumoto (*79*) | 131 | 3.4 (2.0 – 5.7)‡ | 64 | 1.6 (1.2 – 2.2)‡ |
| Sasaki (*85*) | 39 | 4.42 (0.60 – 23.49)* | 44 | 3.01 (0.72 – 10.62)* |

*Abbreviations: SD, standard deviation; *, range; †, IQR (interquartile range); ‡, Q1-Q3 (quartile 1 and 3, respectively)*

## Additional file 2: Table S9. [18F]FDG uptake of the St. Gallen surrogate intrinsic subtypes

|  | **Luminal A** | | **Luminal B** | | **LA/LB** | | **LB HER2-negative** | | **LB HER2-positive** | | **HER2-positive** | | **TNBC** | |
| --- | --- | --- | --- | --- | --- | --- | --- | --- | --- | --- | --- | --- | --- | --- |
| **Study** | **n** | **values** | **n** | **Values** | **n** | **Values** | **n** | **Values** | **n** | **Values** | **n** | **Values** | **n** | **Values** |
| *Mean and SD* | | | | | | | | | | | | | | |
| AbdElaal (*17*) | 10 | 5.88±4.90 | 19 | 10.93±5.55 | - | - | - | - | - | - | 4 | 8.03±5.24 | 7 | 19.19±11.86 |
| Abubakar (*18*) | 23 | 7.75±4.30 | 90 | 10.01±5.34 | - | - | - | - | - | - | 45 | 11.27±5.20 | 55 | 15.26±5.69 |
| Ahn (*19*) | 223 | 3.22±2.15 | 82 | 4.98±2.64 | - | - | - | - | - | - | - | - | - | - |
| Akin (*20*) | 22 | 5.32±3.86 | 12 | 10.46±6.89 | - | - | - | - | - | - | 6 | 6.62±3.99 | 15 | 11.4±11.99 |
| Arslan (*21*) | 18 | 9.9±7.4 | 27 | 12.9±6.6 | - | - | - | - | - | - | 19 | 16.4±11.6 | 26 | 17.7±14.2 |
| Arslan (*22*) | 307 | 10.2±7.2 | 107 | 12.3±6.4 | - | - | - | - | - | - | 35 | 15.0±8.9 | 53 | 17.6±14.2 |
| Bitencourt (*25*) | 17 | 3.5±3.5 | 27 | 4.9±3.9 | - | - | - | - | - | - | 5 | 4.8±3.7 | 9 | 11.9±6.7 |
| Garcia Vicente (*30*) | 23 | 4.7±3.8 | - | - | - | - | 69 | 6.2±4.6 | 35 | 8.4±6.0 | 21 | 8.1±6.7 | 46 | 9.9±5.9 |
| Keam (*38*) | - | - | - | - | 40 | 6.3±3.3 | - | - | - | - | 12 | 6.6±3.2 | 26 | 9.8±5.3 |
| Kitajima (*14*) | 87 | 3.41±2.07 | - | - | - | - | 111 | 5.17±3.52 | 31 | 6.57±3.84 | 12 | 6.6±3.2 | 53 | 6.97±4.17 |
| Koo (*16*) | 334 | 4.69±3.45 | 66 | 6.51±4.18 | - | - | - | - | - | - | 60 | 7.44±4.73 | 92 | 9.83±6.03 |
| Kwon (*42*) | - | - | - | - | - | - | - | - | 47 | 3.95±3.61 | 24 | 4.18±2.53 | 36 | 5.18±5.29 |
| Lee (*44*) | 38 | 4.5±2.3 | - | - | - | - | 72 | 7.2±4.9 | 21 | 7.2±4.3 | 30 | 10.2±5.5 | 22 | 8.8±7.1 |
| Miyake (*46*) | 33 | 4.4±2.2 | - | - | - | - | 32 | 7.7±4.0 | 9 | 7.3±4.7 | 2 | 10.45±1.35 | 13 | 9.1±5.4 |
| Morawitz (*47*) | 9 | 2.74±0.98 | 36 | 4.37±2.73 | - | - | - | - | - | - | 1 | 1.6 | 6 | 9.12±4.11 |
| Noda (*49*) | 53 | 4.4±3.0 | 6 | 4.2±3.0 | - | - | - | - | - | - | 6 | 10.1±4.9 | 4 | 5.5±0.8 |
| Orsaria (*50*) | 21 | 3.3±2.0 | - | - | - | - | - | - | - | - | - | - | 4 | 6.8±2.2 |
| Ozer (*51*) | 26 | 4.24±1.71 | - | - | - | - | 28 | 7.08±2.78 | 14 | 4.05±3.54 | 10 | 5.17±3.59 | 12 | 10.95±0.41 |
| Sengoz (*55*) | 27 | 4.64±2.45 | - | - | - | - | 61 | 6.48±3.73 | 32 | 6.48±3.59 | 16 | 7.13±3.17 | 11 | 8.14±3.71 |
| Wu (*61*) | 6 | 6.08±3.75 | 15 | 5.36±2.64 | - | - | - | - | - | - | 5 | 13.88±3.89 | 12 | 9.3±4.11 |
| Yildirim (*62*) | - | - | - | - | 14 | 5.78±4.97 | - | - | 31 | 8.41±6.30 | - | - | 5 | 11.43±4.09 |
| *Median and (interquartile) range* | | | | | | | | | | | | | | |
| Akdeniz (*64*) | 31 | 4.4  (2.1-36.6)* | 125 | 7.2  (1.1-36.4)* | - | - | - | - | - | - | 29 | 11.5  (2.7-37.8)* | 27 | 9.7  (3.9-48.0)* |
| Can (*66*) | 40 | 4.3  (0.9-20.1)* | 56 | 4.1  (0.8-17.9)* | - | - | - | - | - | - | 12 | 3.1  (1.2-11.9)* | 21 | 4.1  (1.4-18.0)* |
| Has Simsek (*71*) | 75 | 8.0  (4.2-13.0)‡ | - | - | - | - | 149 | 10.0  (6.0-14.0)‡ | 34 | 12.1  (8.4-17.8)‡ | 22 | 12.1  (9.0-16.0)‡ | 76 | 13.5  (8.0-15.5)‡ |

*Table S9. Continued.*

| Ito (*75*) | - | - | - | - | 113 | 3.1  (2.3-5.5)‡ | - | - | - | - | 12 | 7.4  (3.8-12.1)‡ | 13 | 8.8  (5.3-13.2)‡ |
| --- | --- | --- | --- | --- | --- | --- | --- | --- | --- | --- | --- | --- | --- | --- |
| Masumoto (*79*) | 52 | 1.5  (1.1-2.1)‡ | 98 | 3.0  (1.9-4.6)‡ | - | - | - | - | - | - | 28 | 4.2  (1.7-6.4)‡ | 17 | 4.5  (2.6-6.6)‡ |
| Önner (*81*) | 31 | 5.22  (1.48-21.62)* | - | - | - | - | - | - | - | - | - | - | 14 | 17.05  (7.72-42.88)* |
| Sasada (*84*) | 182 | 2.8  (1.8-4.1)‡ | 318 | 4.0  (2.7-6.0)‡ | - | - | - | - | - | - | 316 | 5.6  (3.7-8.5)‡ | 259 | 7.2  (5.0-9.3)‡ |
| Tural (*86*) | 34 | 8.2  (2.1-18.2)* | 19 | 10.1  (3.5-19.69)* | - | - | - | - | - | - | 9 | 14.0  (4.1-22.9)* | 11 | 14.4  (6.6-23.3)* |

*Abbreviations: SD, standard deviation; *, range; †, IQR (interquartile range); ‡, Q1-Q3 (quartile 1 and 3, respectively)*

## Additional file 2: Table S10. [18F]FDG uptake of simplified clinical subtypes

|  | **ER-positive/HER2-negative** | | **HER2-positive** | | **TNBC** | |
| --- | --- | --- | --- | --- | --- | --- |
| **Study** | **n** | **values** | **n** | **Values** | **n** | **Values** |
| *Mean and SD* | | | | | | |
| Basu (*24*) | 59 | 2.68 ± 1.90 | - | - | 18 | 7.27 ± 5.6 |
| Groheux (*31*) | 84 | 6.2 ± 3.6 | 33 | 7.0 ± 4.5 | 54 | 9.8 ± 6.2 |
| Humbert (*33*) | 110 | 7.8 ± 5.4 | 82 | 8.7 ± 4.5 | 60 | 13.7 ± 8.4 |
| Payan (*52*) | 81 | 8.0 ± 5.0 | 79 | 10.0 ± 5.1 | 57 | 13.8 ± 6.8 |
| Straver (*57*) | 19 | 5.40 ± 3.53 | 11 | 5.90 ± 3.94 | 7 | 11.10 ± 5.46 |
| *Median and (interquartile) range* | | | | | | |
| Ege Aktas (*70*) | 32 | 6.9 (5.1 – 9.6)‡ | 25 | 11.2 (6.2 – 12.1)‡ | 8 | 12.1 (8.0 – 14.6)‡ |
| Higuchi (*73*) | 261 | 2.9 (1.7 – 5.1)‡ | 36 | 4.36 (2.73 – 9.08)‡ | 51 | 6.30 (3.59 – 8.22)‡ |
| Koolen (*78*) | 105 | 5.5 (4.9)† | 53 | 6.2 (4.8)† | 56 | 10.8 (9.8)† |

*Abbreviations: SD, standard deviation; *, range; †, IQR (interquartile range); ‡, Q1-Q3 (quartile 1 and 3, respectively)*

## Additional file 2: Table S11. [18F]FDG uptake of TNBC versus non-TNBC

|  | **TNBC** | | **Non-TNBC** | |
| --- | --- | --- | --- | --- |
| **Study** | **n** | **values** | **n** | **Values** |
| *Mean and SD* | | | | |
| Baba (*23*) | 11 | 9.01 ± 6.78 | 46 | 5.83 ± 4.34 |
| Chang (*27*) | 4 | 5.97 ± 4.22 | 28 | 4.44 ± 2.94 |
| Ekmekcoiglu (*28*) | 18 | 13.91 ± 5.52 | 107 | 9.64 ± 7.05 |
| Jo (*35*) | 32 | 11.4 ± 6.3 | 104 | 7.3 ± 5.5 |
| Keam (*38*) | 26 | 9.8 ± 5.3 | 52 | 6.4 ± 3.2 |
| Kim (*39*) | 20 | 5.3 ± 2.5 | 71 | 3.8 ± 2.8 |
| Kitajima (*41*) | 44 | 6.44 ± 3.49 | 172 | 5.42 ± 3.85 |
| Liu (*45*) | 41 | 12.70 ± 9.36 | 232 | 9.78 ± 5.68 |
| Nakajima (*48*) | 12 | 11.1 ± 10.4 | 81 | 5.5 ± 3.5 |
| Orsaria (*50*) | 4 | 6.8 ± 2.2 | 46 | 4.2 ± 2.9 |
| Ravina (*54*) | 38 | 10.19 ± 7.03 | 72 | 7.77 ± 4.75 |
| Tchou (*58*) | 22 | 7.0 ± 5.5 | 18 | 2.8 ± 2.5 |
| Ugurluer (*60*) | 11 | 7.20 ± 4.31 | 128 | 6.10 ± 4.53 |
| *Median and (interquartile) range* | | | | |
| An (*65*) | 17 | 10.2 (3.6 – 21.0)* | 50 | 5.3 (1.3 – 16.2)* |
| Cochet (*69*) | 10 | 11.9 (10.2 – 14.2)‡ | 30 | 6.4 (5.1 – 9.0)‡ |
| Groheux (*13*) | 35 | 9.2 (6.1 – 14.6)‡ | 96 | 5.8 (3.7 – 8.2)‡ |
| Heudel (*72*) | 12 | 7.0 (2.7 – 19.5)* | 33 | 2.8 (0.7 – 11.7)* |
| Kaida (*76*) | 13 | 4.10 (1.75 – 9.10)* | 80 | 2.7 (1.1 – 9.1)* |
| Sanli (*83*) | 9 | 15.00 (2.09 – 39.00)* | 70 | 11.59 (2.00 – 39.00)* |

*Abbreviations: SD, standard deviation; *, range; †, IQR (interquartile range); ‡, Q1-Q3 (quartile 1 and 3, respectively)*

## Additional file 2: Table S12. QUADAS-2 table

| Author | Risk of bias | | | | Applicability concerns | | |
| --- | --- | --- | --- | --- | --- | --- | --- |
|  | Patient selection | Index test | Reference standard | Flow and timing | Patient selection | Index test | Reference standard |
| *Mean and SD* | | | | | | | |
| AbdElaal (*17*) | Low | Low | Unclear | Low | Low | Low | Low |
| Abubakar (*18*) | Low | Low | Unclear | High | Low | Low | Low |
| Ahn (*19*) | Low | Unclear | Low | Low | High | Low | Low |
| Akin (*20*) | Low | Unclear | Low | Low | Low | Low | Low |
| Arslan (*21*) | Low | Unclear | Unclear | Low | Low | Low | Low |
| Arslan (*22*) | Unclear | High | Unclear | Low | Low | Low | Low |
| Baba (*23*) | Low | Unclear | Unclear | Low | Low | Low | Low |
| Basu (*24*) | High | Low | Unclear | High | Low | Low | Low |
| Bitencourt (*25*) | Low | Low | Unclear | High | Low | High | Low |
| Catalano (*26*) | Low | Low | Low | Low | Low | Low | Low |
| Chang (*27*) | Low | Unclear | Low | Low | Low | Low | Low |
| Ekmekcioglu (*28*) | Low | Unclear | Low | High | Low | Low | Low |
| García Vicente (*29*) | Low | Low | Low | Low | Low | Low | Low |
| García Vicente (*30*) | Low | Low | Low | Low | Low | Low | Low |
| Gil-Rendo (*12*) | Low | Low | Low | High | High | Low | Low |
| Groheux (*31*) | Low | Low | Low | Low | Low | Low | Low |
| Humbert (*32*) | Low | Unclear | Low | Low | Low | Low | Low |
| Humbert (*33*) | Low | High | Unclear | Low | Low | Low | Low |
| Jeong (*34*) | Low | Low | Low | Low | Low | Low | Low |
| Jo (*35*) | Low | Low | Unclear | Low | Low | Low | Low |
| Jung (*36*) | High | High | Low | High | High | Low | Low |
| Kadoya (*37*) | Low | Unclear | Low | Low | Low | Low | Low |
| Keam (*38*) | Low | Unclear | Low | Low | Low | Low | Low |
| Kim (*39*) | Low | Unclear | Low | Low | Low | Low | Low |
| Kim (*87*) | Low | High | Low | Low | High | Low | Low |
| Kitajima (*14*) | Low | Low | Low | Low | Low | Low | Low |
| Kitajima (*41*) | Low | Low | Low | Low | Low | Low | Low |
| Koo (*16*) | Low | High | Low | Low | Low | Low | Low |
| Kwon (*42*) | Low | Low | Low | Low | Low | Low | Low |
| Lee (*43*) | Low | High | Low | Low | High | Low | Low |
| Lee (*44*) | Low | Low | Low | Low | Low | Low | Low |
| Liu (*45*) | Low | Low | Unclear | Low | Low | Low | Low |
| Miyake (*46*) | Low | Low | Low | Low | Low | Low | Low |
| Morawitz (*47*) | Low | Unclear | Unclear | High | Low | Low | Low |
| Nakajima (*48*) | Low | Low | Unclear | Low | High | Low | Low |
| Noda (*49*) | Low | Low | Low | Low | Low | Low | Low |
| Orsaria (*50*) | Low | Low | Low | Low | Low | Low | High |
| Ozer (*51*) | Low | Low | Low | Low | Low | Low | Low |
| Payan (*52*) | Low | Unclear | Low | Low | Low | Low | Low |
| Qu (*53*) | Low | Low | Low | Low | Low | Low | Low |
| Ravina (*54*) | Low | Low | Unclear | High | Low | Low | Low |
| Sengoz (*55*) | Low | Low | Low | High | Low | Low | Low |
| Song (*56*) | Low | High | Low | Low | Low | Low | Low |
| Straver (*57*) | Low | Low | Unclear | Low | Low | Low | Low |

*Table S12. Continued.*

| Tchou (*58*) | High | Low | Unclear | High | Low | Low | Low |
| --- | --- | --- | --- | --- | --- | --- | --- |
| Ueda (*59*) | Low | Unclear | Low | Low | Low | Low | Low |
| Ugurluer (*60*) | Low | Unclear | Unclear | Low | Low | Low | Low |
| Wu (*61*) | Low | Low | Unclear | Low | Low | Low | Low |
| Yildirim (*62*) | Low | Low | Unclear | High | Low | Low | Low |
| Yoon (*63*) | Low | Low | Low | Low | Low | Low | Low |
| *Median and (interquartile) range* | | | | | | | |
| Akdeniz (*64*) | Low | High | Low | Low | Low | Low | Low |
| An (*65*) | Unclear | Low | Unclear | Low | Low | Low | Low |
| Can (*66*) | Low | Low | Low | Low | Low | Low | Low |
| Cerci (*67*) | Low | Unclear | Unclear | Low | Low | Low | Low |
| Choi (*68*) | Low | High | Low | Low | Low | Low | Low |
| Cochet (*69*) | Low | Unclear | Unclear | Low | Low | High | Low |
| Ege Aktas (*70*) | Low | Unclear | Low | Low | Low | Low | Low |
| Groheux (*13*) | Low | Low | Low | Low | Low | Low | Low |
| Has Simsek (*71*) | Low | Low | Low | High | Low | Low | Low |
| Heudel (*72*) | Low | Unclear | Low | Low | Low | Low | Low |
| Higuchi (*73*) | Low | Unclear | Low | Low | Low | Low | Low |
| Ito (*75*) | Low | Unclear | Low | Low | Low | Low | Low |
| Iqbal (*74*) | Low | High | Low | Low | High | Low | Low |
| Kaida (*76*) | Low | Low | Low | Low | Low | Low | Low |
| Karan (*77*) | Low | Low | Low | Low | Low | Low | Low |
| Koolen (*78*) | Low | Low | Low | Low | Low | Low | Low |
| Masumoto (*79*) | Low | Low | Low | Low | Low | Low | Low |
| Moon (*80*) | High | Unclear | Unclear | Low | Low | Low | Low |
| Önner (*81*) | Low | Low | Low | Low | High | Low | Low |
| Ozen (*82*) | Low | Unclear | Low | Low | Low | Low | Low |
| Sanli (*83*) | Low | Unclear | Low | Low | Low | Low | Low |
| Sasada (*84*) | Low | Low | Low | Low | Low | Low | High |
| Sasaki (*85*) | Unclear | Low | Low | Low | Low | Low | Low |
| Tural (*86*) | Low | Low | Low | Low | Low | Low | Low |
